# Changing the Reactivity of Zero‐ and Mono‐Valent Germanium with a Redox Non‐Innocent Bis(silylenyl)carborane Ligand

**DOI:** 10.1002/anie.202103769

**Published:** 2021-06-01

**Authors:** Shenglai Yao, Arseni Kostenko, Yun Xiong, Christian Lorent, Ales Ruzicka, Matthias Driess

**Affiliations:** ^1^ Department of Chemistry: Metalorganics and Inorganic Materials Technische Universität Berlin Strasse des 17. Juni 135, Sekr. C2 10623 Berlin Germany; ^2^ Department of Chemistry, Physical and Biophysical Chemistry Technische Universität Berlin Strasse des 17. Juni 135, Sekr. PC14 10623 Berlin Germany; ^3^ Department of General and Inorganic Chemistry Faculty of Chemical Technology University of Pardubice Studentska 573 53210 Pardubice Czech Republic

**Keywords:** carboranes, germanium, germylone, redox non-innocent ligands, tetrylones

## Abstract

Using the chelating C,C′‐bis(silylenyl)‐ortho‐dicarborane ligand, 1,2‐(RSi)_2_‐1,2‐C_2_B_10_H_10_ [R=PhC(NtBu)_2_], leads to the monoatomic zero‐valent Ge complex (“germylone”) **3**. The redox non‐innocent character of the carborane scaffold has a drastic influence on the reactivity of **3** towards reductants and oxidants. Reduction of **3** with one molar equivalent of potassium naphthalenide (KC_10_H_8_) causes facile oxidation of Ge^0^ to Ge^I^ along with a two‐electron reduction of the C_2_B_10_ cluster core and subsequent Ge^I^‐Ge^I^ coupling to form the dianionic bis(silylene)‐supported Ge_2_ complex **4**. In contrast, oxidation of **3** with one molar equivalent of [Cp_2_Fe][B{C_6_H_3_(CF_3_)_2_}_4_] as a one‐electron oxidant furnishes the dicationic bis(silylene)‐supported Ge_2_ complex **5**. The Ge^0^ atom in **3** acts as donor towards GeCl_2_ to form the trinuclear mixed‐valent Ge^0^→Ge^II^←Ge^0^ complex **6**, from which dechlorination with KC_10_H_8_ affords the neutral Ge_2_ complex **7** as a diradical species.

## Graphical abstract

The bis(silylenyl) *ortho*‐dicarborane‐supported zero‐valent Ge complex (germylone) **1** undergoes facile dimerization with Ge−Ge bond formation upon one‐electron oxidation and reduction to furnish the bis(silylene)‐supported dicationic and dianionic [Ge^I^–Ge^I^] complexes **2** and **3**, respectively. A neutral complex **4** bearing a Ge_2_^2+^ dumbbell embedded in a shell of two radical‐anionic di(silylenyl)dicarborane ligands could be synthesized.

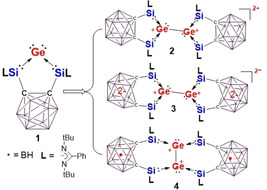

Tetrylones have emerged recently which represent a new class of molecules featuring monoatomic, zero‐valent Group 14 elements directly stabilized by two σ‐donor ligands (L) through Lewis donor–acceptor interaction (L:→:E:←:L, E=C, Si, Ge, Sn, Pb).[^1^, ^2^, ^3^, ^4^, ^5^, ^6^, ^7^, ^8^] Since the central atoms of tetrylones retain their four valence electrons as two lone pairs, this type of species have been considered as soluble “allotrope” of the respective elements.^[9]^ Owing to the peculiar bonding situation and the zero‐valent nature of the central atoms, tetrylones may exhibit a versatile reactivity with access to new types of low‐valent Group 14 element compounds. Utilizing iminopyridines,[^10^, ^11^] iminocarbenes,[^12^, ^13^] carbenes,[^14^, ^15^, ^16^, ^17^, ^18^, ^19^] silylenes,[^20^, ^21^, ^22^, ^23^, ^24^] and germylenes[^25^, ^26^] as supporting σ‐donor ligands L, various examples of carbones,[^14^, ^15^] silylones,[^16^, ^18^, ^20^, ^22^, ^23^, ^24^, ^26^] germylones,[^11^, ^12^, ^13^, ^17^, ^19^, ^21^, ^25^] and stannylones^[10]^ have been synthesized and structurally characterized. However, compared to carbenes and related metallylenes, the number of isolable tetrylones is still limited and their reactivity much less explored. The reactivity of tetrylones documented so far is dominated by their nucleophilic ability to form Lewis adducts and to undergo oxidative addition reactions.[^1^, ^4^]

Recently, we showed that the chelating bis(NHSi) (NHSi=*N*‐heterocyclic silylene) ligand **1** (Scheme 1), 1,2‐bis(RSi)_2_‐1,2‐dicarba‐*closo*‐dodecaborane(12), [R=PhC(N*t*Bu)_2_], can act as a redox non‐innocent dicarborane scaffold towards main‐group elements and transition‐metals.[^27^, ^28^] It enabled us to develop a zero‐valent monosilicon complex (“silylone”)^[22]^ in which the Si^0^ atom can undergo [Si^I^‐Si^I^] bond formation upon one‐electron reduction. Herein, we report the synthesis of the first redox‐active germylone **3** (Scheme 1) and its reactivity applying different redox reaction conditions which led to a series of new bis(NHSi)dicarborane‐supported Ge_2_^2+^ complexes.

**Scheme 1:**
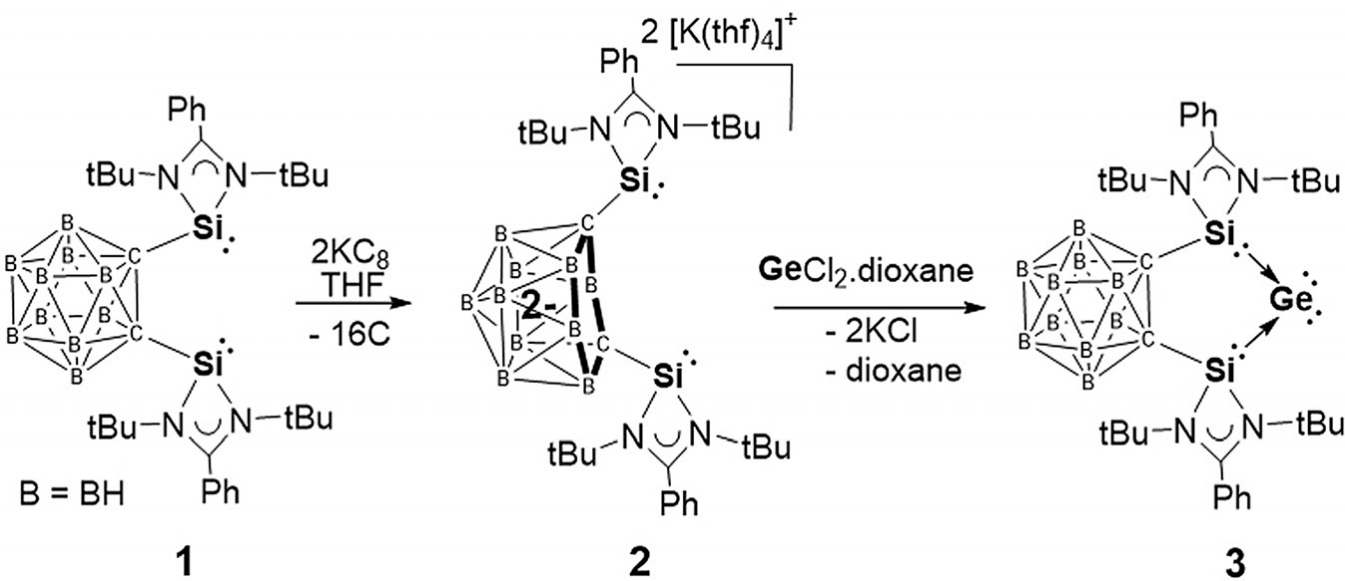
Synthesis of the germylone **3** from C,C′‐bis(silylenyl) dicarborane ligand **1** via **2**.

Treatment of the dipotassium bis(NHSi) *nido*‐dicarboranate precursor **2**, prepared in situ from **1** and two molar equivalents of C_8_K in THF (Scheme 1),^[22]^ with one molar equivalent of GeCl_2_–dioxane at room temperature leads to formation of the new germylone **3**. Complex **3** was isolated as a brown‐red powder in 64 % yields. The molecular structure of **3** has *C*_2*v*_ symmetry with a planar five‐membered C_2_Si_2_Ge ring perpendicular to both four‐membered CN_2_Si rings (Figure 1). The two‐coordinate Ge center features two almost identical Ge–Si distances [Ge1–Si1: 2.2896(5) and Ge1–Si2: 2.2846(5) Å], slightly shorter than those observed for the xanthene‐based bis(silylene)‐stabilized germylone [2.3147(9) and 2.23190(9) Å].^[21]^ Notably, the Si‐Ge‐Si angle of 80.59(2)° in **3** is much more acute than in the xanthene‐based germylone [102.87(3)°] and even smaller than that of bis(NHC)‐supported germylone [86.6(1)°].^[17]^ The C1–C2 distances [1.671(2) Å] in **3** is slightly shorter than that in **1** [1.71 Å],^[27]^ suggesting that the silylene donors are bridged by a *closo*‐C_2_B_10_ cluster core.

**Figure 1:**
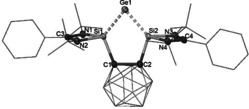
Molecular structure of **3**.^[31]^ Thermal ellipsoids are set at the 50 % probability level. H atoms are omitted for clarity.

Akin to the analogous silylone with the same bis(silylene) ligand **1**,^[22]^ the HOMO and the HOMO‐1 in **3** correspond to the germylone lone pairs with π‐ and σ‐symmetry, respectively (Figure 2). NBO analysis shows that in **3** the Ge^0^ π‐symmetry lone pair, with occupancy of 1.11 el., exhibits strong donor‐acceptor interaction with the low‐valent 3p orbitals of the amidinato‐silylene Si atoms (175.2 kcal mol^−1^). The π‐symmetry lone pair with occupancy of 1.80 el. mainly interacts with the low‐valent sp^3^ orbitals of the amidinato‐silylene Si atoms (25.7 kcal mol^−1^, Figure S18 in the Supporting Information).

**Figure 2:**
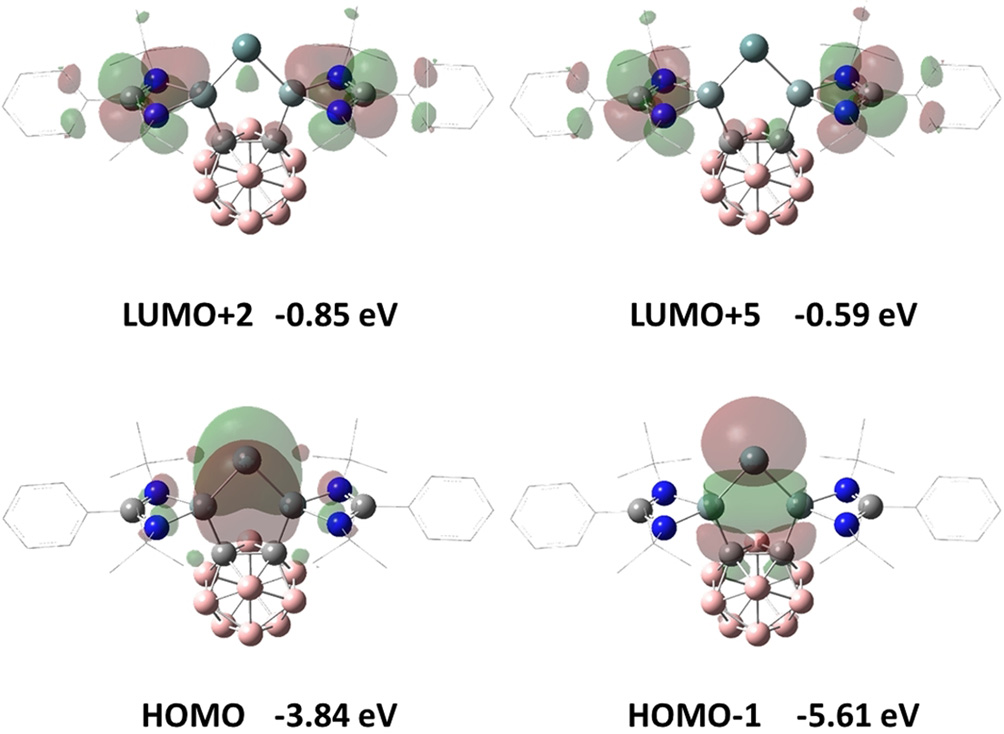
Selected frontier orbitals of **3**. Hydrogen atoms are omitted for clarity. LUMO+1, LUMO+3, LUMO+4 (not shown) correspond to the phenyl π* orbitals.

We investigated the redox behavior of **3** with cyclic voltammetry, which shows multiple irreversible redox events (Figure S5). The complexity of the latter is presumably due to a facile oxidation‐state change of the Ge center and electronic structure variation involving the redox non‐innocent amidinato ligand and the C_2_B_10_ cluster core. In order to achieve a controllable single‐electron reduction of **3**, we conducted the reaction with one molar equiv of potassium naphthalenide, which, in fact, led to the isolation of the Ge^I^–Ge^I^ coupling product **4** as a dark red crystalline solid in 95 % yields (Scheme 2).

**Scheme 2:**
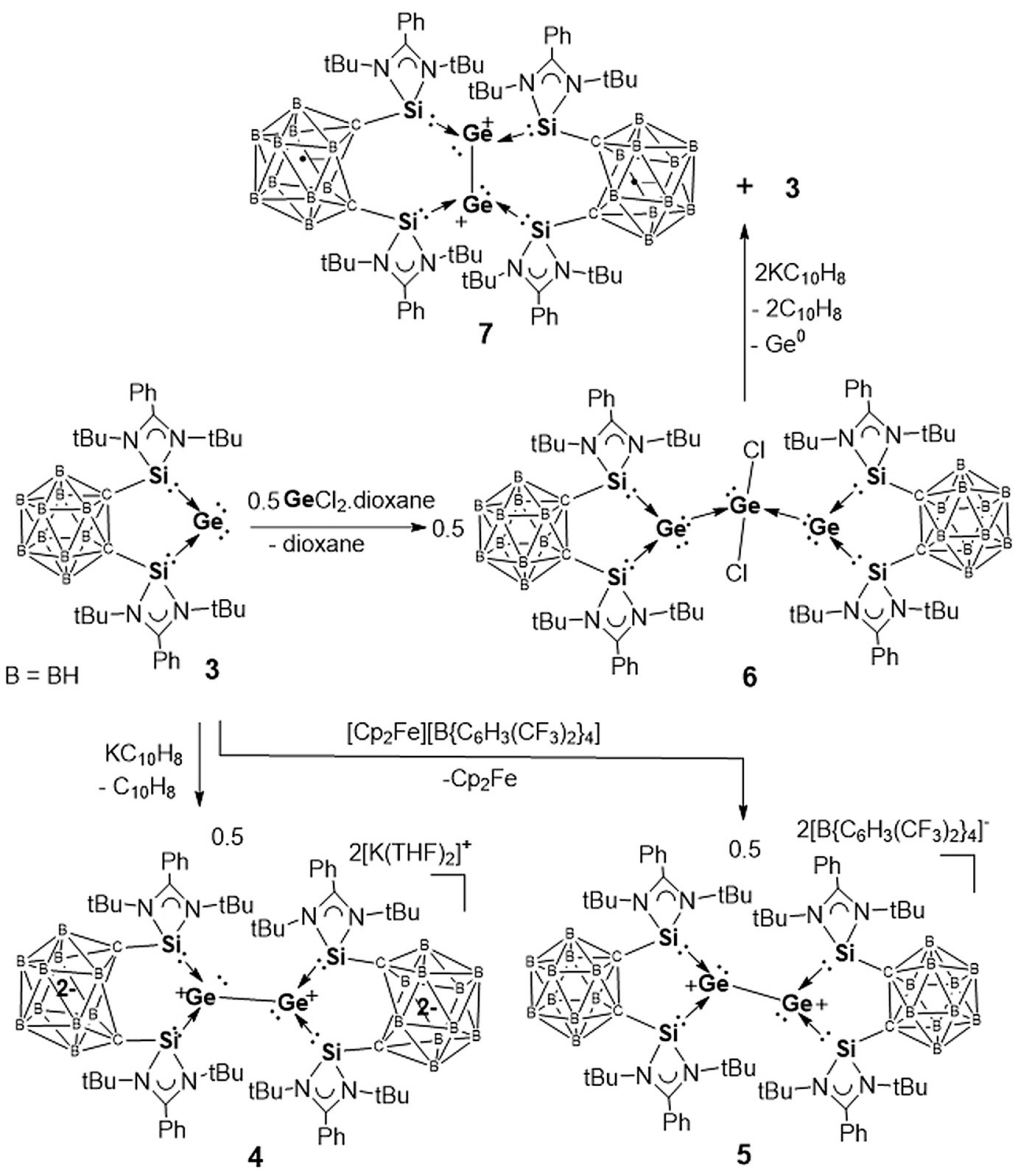
Synthesis of the unprecedented [Ge^I^‐Ge^I^] complexes **4**, **5**, and **7** from the germylone **3**.

The crystal structure of **4** reveals a one‐dimensional ionic polymer in which the dianionic units feature a Ge–Ge core coordinated by one bis(silylenyl)‐*nido*‐dicarborane ligand at each Ge site (Figure 3) and two K cations linked to two dicarborane units via agostic interaction with the B−H bonds of the C_2_B_10_H_10_ clusters. Both Ge atoms possess a lone pair of electrons and are three‐coordinated. The Ge–Si distances [Ge1–Si1 2.4294(8) and Ge1–Si2 2.4105(8) Å] are significantly longer than those Ge–Si distances in **3** [2.2896(5) and 2.2846(5) Å]. Representing the first bis(silylene)‐supported [Ge^I^–Ge^I^]^2+^, compound **4** features a Ge–Ge distance of 2.5161(6) Å which is shorter than that in a bis(NHC) borate‐stabilized [Ge^I^–Ge^I^]^2+^ [2.673(1) Å],^[29]^ but comparable to the value in the amidinato digermylene {[PhC(N*t*Bu)_2_]Ge:}_2_ [2.569(5) Å].^[30]^ The C1⋅⋅⋅C2 distance of 2.68 Å indicates that the two silylene units in **4** are bridged by a dianionic *nido*‐C_2_B_10_ cluster core.

**Figure 3:**
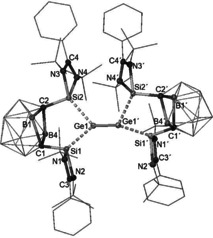
Molecular structure of the dianion unit of **4**.^[31]^ Thermal ellipsoids are set at the 30 % probability level. H atoms are omitted for clarity.

The Ge–Ge coupling of **4** is reminiscent of the formation of the analogous [Si^I^–Si^I^]^2+^ complex.^[22]^ According to DFT calculations reported previously for the latter Si_2_ homologue, the one‐electron reduction transforms the *closo*‐C_2_B_10_H_10_ bridge to the opened dianionic *nido*‐cluster and the Ge^0^ center to a Ge^I^ radical (Figure 4), which undergoes Ge^I^–Ge^I^ coupling to furnish **4** as an isolable product. The electronic structure of **4** closely resembles that of the aforementioned Si_2_ homologue (Figure S20).^[22]^

**Figure 4:**
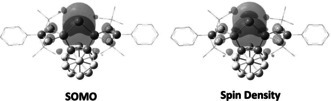
Singly occupied molecular orbital (SOMO) and Mulliken spin density of the proposed intermediate radical cation of **3**.

To investigate the one‐electron oxidation, **3** was allowed to react with one molar equiv of [Cp_2_Fe][B{C_6_H_3_(CF_3_)_2_}_4_] in THF at room temperature, which led to the isolation of a new [Ge^I^–Ge^I^] coupling compound **5** as an orange solid in 92 % yield (Scheme 2). This product is also insoluble in diethyl ether, but well soluble in THF. The ^1^H NMR spectrum of **5** in [D_8_]THF exhibits only one singlet at *δ*=1.46 ppm for the *t*Bu groups, while the ^29^Si{^1^H} NMR spectrum shows a broad resonance at *δ*=68.0 ppm.

The single‐crystal structure of **5** reveals a separate ion pair with a Ge_2_‐containing dication and two borate counteranions (Figure 5). Similar to the structure of **4**, both Ge centers in **5** adopt a trigonal‐pyramidal coordination geometry and the Ge–Ge distance of 2.5468(3) Å is close to that in **4** [2.5161(6) Å]. A marked metric difference between the dication in **5** and the dianion in **4** represents the carborane_C−C distance [**5**: ca.1.67 Å vs. **4**: 2.68 Å], due to the presence of a *nido*‐ core in **4** vs. a *closo*‐C_2_B_10_H_10_ cluster in **5**. We propose that the formation of **5** upon oxidation of **3** is achieved by a one‐electron transfer from the HOMO of **3** (Figure 2), forming the corresponding Ge^I^ radical cation intermediate in which 74 % of the spin density resides on the Ge atom (Figure 4), and subsequent radical coupling. Similar to the dianion in **4**, the dication in **5** contains a Ge−Ge bond with a Wiberg Bond Index (WBI) of 0.86, and retains one σ‐symmetry lone pair on each of the Ge atoms, as confirmed by the Natural Bond Orbital (NBO) analysis (Figure S19).

**Figure 5:**
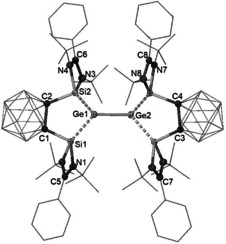
Molecular structure of the dianion of **5**.^[31]^ Thermal ellipsoids are set at the 30 % probability level. H atoms are omitted for clarity.

To explore the coordination ability of the Ge^0^ center, compound **3** was allowed to react with GeCl_2_—dioxane (Scheme 2). The reaction yields exclusively compound **6** as a yellow solid regardless of the ratio of reactants. **6** is insoluble in common aprotic organic solvent. Its ^29^Si solid‐state NMR spectrum shows a resonance at *δ*=56.0 ppm. The molecular structure of **6** reveals a seesaw coordination geometry for the central Ge center with both chloride atoms located at the axial positions (Cl1‐Ge3‐Cl2: 163.2°), suggesting that one of the equatorial position is occupied by a lone pair (Figure 6). Compound **6** can be viewed as a Ge^0^_2_Ge^II^ adduct, and each Ge^0^ atom features still a stereochemically active lone pair as indicated by its pyramidal coordination geometry (sum of bond angles each ca. 295°). The Ge–Ge distances [Ge1–Ge3 2.412(4) Å] and Ge2–Ge3 [2.416(5) Å] are considerably shorter than those Ge−Ge bonds in **4** [2.5161(6) Å] and **5** [2.5468(3) Å], suggesting a relatively strong Ge–Ge interaction in **6**.

**Figure 6:**
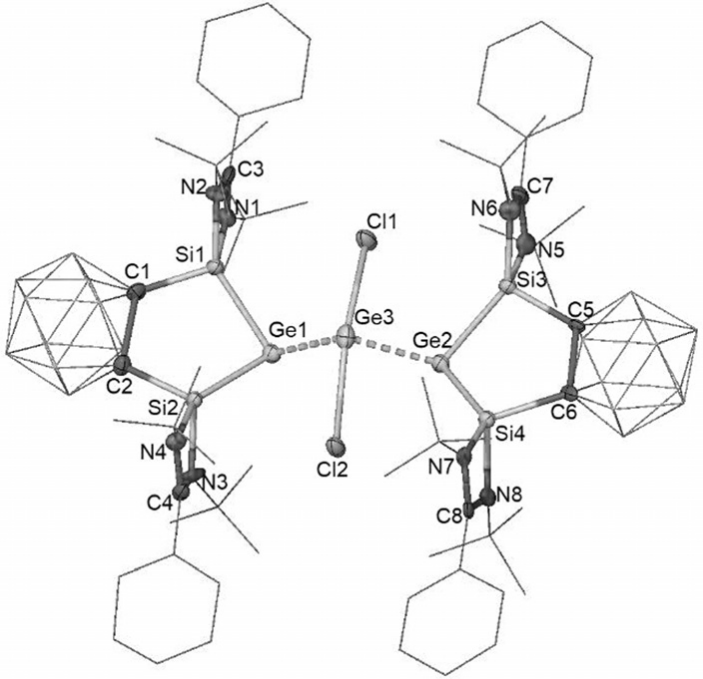
Molecular structure of the dianion of **6**.^[31]^ Thermal ellipsoids are set at the 30 % probability level. H atoms are omitted for clarity.

Interestingly, complex **6** can serve as a precursor for the novel bis(NHSi)‐supported neutral Ge_2_ complex **7**, which is obtained as a minor isolable product (28 % yields) along with germylone **3** (58 % yields) from dechlorination of **6** with two molar equivs of potassium naphthalenide (scheme 2). Compound **7** is NMR silent; its molecular structure has been established by single‐crystal X‐ray diffraction analysis (Figure 7). **7** features a Ge_2_ dumbbell coordinated by two bis(silylene) ligands in a side‐on manner. Due to the electronic neutral nature of the Ge_2_^2+^ complex, each of the C_2_B_10_H_10_ clusters carries one negative charge and thus is a radical in keeping with the observed NMR silence of **7**. In agreement with this, the C−C distance in the C_2_B_10_ core in **7** is ca. 2.4 Å, lying closer to the C−C value in **4** (*nido*‐C_2_B_10,_ 2.68 Å) than that in **5** (*closo*‐C_2_B_10_, 1.67 Å). The triplet diradical nature of **7** has been confirmed by its electron paramagnetic resonance spectrum (Figure S12), which exhibits an isotropic signal at *g*=2.004 very similar to the spectral signature of known carborane radical anions.^[28]^ Furthermore, DFT calculations support the triplet diradical assignment. Optimization of **7** in triplet and singlet states leads to geometries in which the triplet state is lower in energy by 15.8 kcal mol^−1^. As shown in Figure 8, the spin density (1.92 e^−^) of **7** in the triplet state is localized at the carborane moieties and NBO analysis shows the Ge–Ge WBI of 0.85. It should be noted, although **7** can be considered as a dimer of germylone **3** and the dimerization reaction is endergonic by 1.8 kcal mol^−1^), no equilibrium between **3** and **7** has been observed, presumably, due to the difference of their coordination modes.

**Figure 7:**
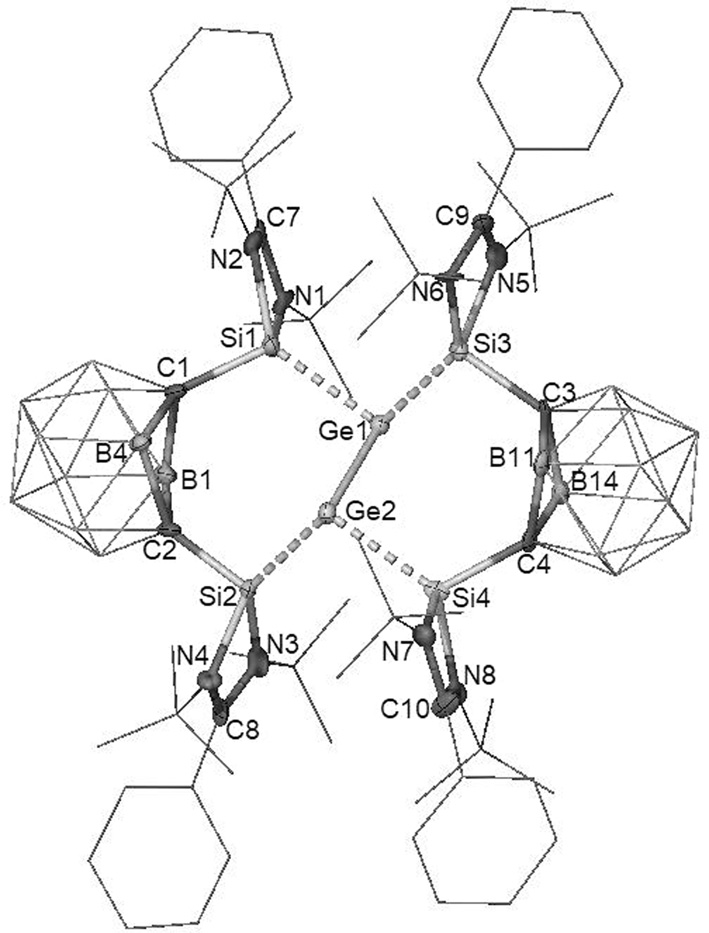
Molecular structure of **7**.^[31]^ Thermal ellipsoids are set at the 30 % probability level. H atoms are omitted for clarity.

**Figure 8:**
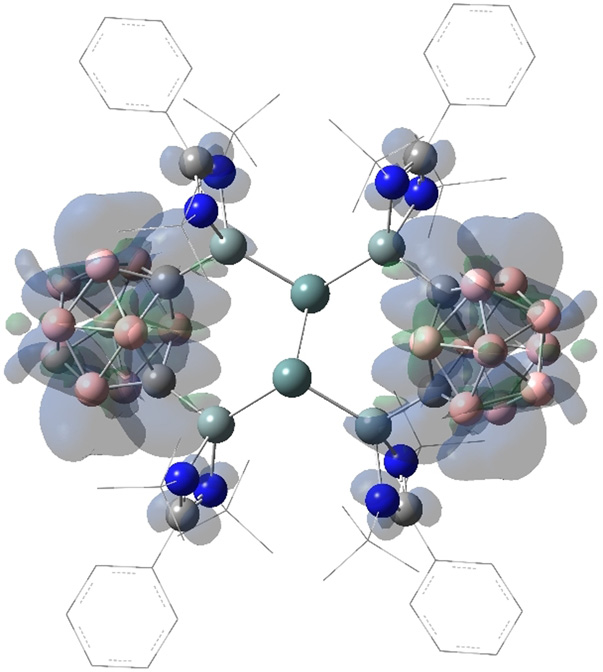
Mulliken spin density of **7**.

In summary, a series of unexpected novel low‐valent Ge_2_ complexes could be synthesized, starting from the monoatomic zero‐valent Ge^0^ complex **3** supported by the redox non‐innocent bis(silylenyl)‐*ortho*‐dicarborane ligand. While its one‐electron reduction affords the bis(silylene)‐supported [Ge^I^–Ge^I^] dianion complex in **4**, the one‐electron oxidation leads to the bis(silylene)‐stabilized [Ge^I^–Ge^I^] dication complex in **5**. Moreover, coordination of two germylone molecules of **3** with one GeCl_2_ allowed the isolation of the mixed‐valent trinuclear Ge complex **6**, which serves as a precursor for the neutral Ge_2_ complex **7** with a triplet diradical ground state.

## Conflict of interest

The authors declare no conflict of interest.

## Supporting information

As a service to our authors and readers, this journal provides supporting information supplied by the authors. Such materials are peer reviewed and may be re‐organized for online delivery, but are not copy‐edited or typeset. Technical support issues arising from supporting information (other than missing files) should be addressed to the authors.

Supplementary
