# Supplementary material for: Changing the Reactivity of Zero‐ and Mono‐Valent Germanium with a Redox Non‐Innocent Bis(silylenyl)carborane Ligand
Source: Angew Chem Int Ed Engl. 2021 Jun 1;60(27):14864–8. doi: 10.1002/anie.202103769 (PMC8252802; doi:10.1002/anie.202103769)
Supplement: Supplementary file 1 — Supplementary [file ANIE-60-14864-s001.pdf]

Angewandte Chemie
Eine Zeitschrift der Gesellschaft Deutscher Chemiker

Supporting Information

Changing the Reactivity of Zero- and Mono-Valent Germanium with a Redox Non-Innocent Bis(silylenyl)carborane Ligand

*Shenglai Yao, Arseni Kostenko, Yun Xiong, Christian Lorent, Ales Ruzicka, and Matthias Driess**

anie_202103769_sm_miscellaneous_information.pdf

Table of Content

A. Experimental Section…………………………………………….………………...…..……... 2

A1 General Considerations……………………………………….……….……….…..…….……2

A2 Single-Crystal X-ray Structure Determination………………………...…...….…………..…..2

A3 EPR Measurement………………………………...…………..……………...………..…..2

A4 Cyclic Voltammetry Measurement……..……………..…………..3

A5 Synthesis and Characterization………………………………….……………..……….…..3

A6 Details of the Single-Crystal X-ray diffraction analyses …………………….……..………12

B Computational Section…………………………...….……………………….…..….……..27

C References……………………………………………….……….….….……….....…….48

A. Experimental Section

A1. General Considerations

All experiments were carried out under dry oxygen-free nitrogen using standard Schlenk techniques or an MBraun glove box fitted with a gas purification and recirculation unit. Solvents were dried by standard methods and freshly distilled prior to use. The starting material, C,C´-bis(silylenyl)-substituted carborane **1** [1] was prepared according to the literature procedures. Germanium(II) chloride dioxane complex was purchased from Sigma-Aldrich. Potassium graphite was prepared by reacting potassium with previously dried graphite in a 1:8 ratio at 160 °C for 2 h under dried nitrogen. The NMR spectra were recorded with Bruker spectrometers ARX200 and AV400 referenced to residual solvent signals as internal standards. Abbreviations: *s* = singlet; *d* = doublet; *t* = triplet; *sept* = septet; *m* = multiplet; *br* = broad. Elemental analyses were performed by the analytical labor service in the Institute of Chemistry, Technical University of Berlin, Germany. High-resolution ESI-MS were measured on a Thermo Scientific LTQ orbitrap XL. IR spectra were measured with a Nicolet iS5 FT-IR-Spectrometer from the company Thermo Scientific.

A2. Single-Crystal X-ray Structure Determination

The crystal was mounted on a glass capillary in per-fluorinated oil and measured in a cold N_2 flow. The data of **3**-**7** were collected on an Oxford Diffraction Supernova, Single source at offset, Atlas at 150 K (Cu- Kα-radiation, λ = 1.5418 Å). The structure was solved by direct method and refined on F^2 with the SHELX-97[2a] software package. The positions of the H atoms were calculated and considered isotropically according to a riding model. In the molecular structure of compound **4** the carborane moiety and one of the *tert*-butyl groups are disordered over two orientations with an occupancy ratio of 0.50:0.50. There is a free but highly disordered THF molecule within the asymmetric unit which was treated using the SQUEEZE routine in PLATON. In the molecular structure of compound **5** several CF_3 groups of the $[B\{C_6H_3(CF_3)_2\}_4]^-$ anions are disordered over two orientations with different occupancy ratios. In the molecular structure of compound **6** part of the amidinato ligands are disordered over two orientations with an occupancy ratio of 0.56:0.44 and the central $Ge(GeCl_2)Ge$ moiety is disordered over two orientations with an occupancy ratio of 0.50:0.50. In addition, several co-crystallized free THF molecules within the asymmetric unit are highly disordered and treated using the SQUEEZE routine in PLATON. In the molecular structure of compound **7** the central Ge atoms are disordered over two orientations with an occupancy ratio of 0.87:0.13. Moreover, several co-crystallized free THF molecules within the asymmetric unit are highly disordered and treated using the SQUEEZE routine in PLATON. CCDC 2070702 (**3**), 2070706 (**4**), 2070703 (**5**), 2070704 (**6**) and 2070705 (**7**) contain the supplementary crystallographic data for this paper. These data can be obtained free of charge from The Cambridge Crystallographic Data Centre via www.ccdc.cam.ac.uk./data_request/cif.

A3. EPR Spectroscopy

The EPR spectrum (Figure S12) was recorded on a Bruker EMXplus spectrometer equipped with an ER 4122 SHQE resonator. Experimental conditions: 1 mW microwave power, microwave frequency: 9.3 GHz, 1 G modulation amplitude, 100 kHz modulation frequency. Simulation of the spectrum was conducted using the MATLAB toolbox EasySpin (version 5.2.25).[2b]

A4. Cyclic Voltammetry Measurement

Cyclic voltammetry (CV) measurements of compound **3** (Figure S5) were performed in a standard three-electrode electrochemical cell having Pt-wire used as an auxiliary electrode, glassy carbon (3 mm diameter) as working electrode and Ag/Ag^+ as a pseudo reference electrode at 295 K using a Biologic SP-150 potentiostat. All cyclic voltammograms were referenced against the Cp_2Fe/Cp_2Fe^+ redox couple (Fc/Fc^+), which was used as an internal standard. 0.3 M tetrabutyl ammonium hexaflurophosphate ($TBAPF_6$) in THF was used as an electrolyte. The iR-drop was determined and compensated by using the impedance measurement technique implemented in the EC-Lab Software V10.37.

A5. Synthesis and Characterization

Synthesis of 3

To a Schlenk tube charged with **1** (0.22 g, 0.33 mmol) and potassium graphite (0.090 g, 0.67 mmol) was added 15 mL THF at room temperature with stirring. After stirring at room temperature for 1.5 h, the resulting suspension was filtered to give an orange solution. The latter solution was then cooled to -40 °C and $GeCl_2$-dioxane$_2$ (0.077 g, 0.33 mmol) was added with stirring. The reaction mixture was allowed to warm to room temperature and stirred further for 2 h. Volatiles were then removed under vacuum and the residue was extracted with Et_2O (3 × 25 mL). Concentrating and cooling the filtrate at -30 °C for 48 h afforded brown-red crystals of **3** with some yellowish precipitate. Recrystallization with Et_2O furnished compound **3** as brown-red crystals. After removing the co-crystallized diethyl ether under vacuum, pure compound **3** was obtained as a brown-red powder (Yield: 0.15 g, 64 %). M.p. 271 ºC (decomp.). ^{1}H NMR (200 MHz, D_8-THF, 298K): δ = 1.31 (s, 36 H, NC(C*H*$_3$)$_3$), 2.3 – 3.2 (vb, 10 BH, very broad and unresolved), 7.40-7.44 (m, 2 H, Ph-*H*), 7.48-7.57 (m, 6 H, Ph-*H*), 7.66-7.69 ppm (m, 2 H, Ph-*H*). ^{13}C{^{1}H} NMR (100 MHz, D_8-THF, 298K): δ = 32.3 (s, NC(*C*H$_3$)$_3$), 56.7 (s, N*C*(CH$_3$)$_3$), 82.4 (s, carborane-C), 128.3, 129.0, 129.2, 131.4, 133.5 (s, Ph-*C*), 176.9 (s, N*C*N). ^{29}Si{^{1}H} NMR (99.49 MHz, D_8-THF, 298 K): δ = 56.5 ppm (*Si*$_2$Ge). ^{11}B{^{1}H} NMR (64 MHz, D_8-THF, 298 K): δ = -14 - 3 ppm (m, vb). ESI-MS: m/z: 735.43276 (calc. 735.43312 $[M+H]^+$); Elemental analysis calcd for $C_{32}H_{56}N_4B_{10}Si_2Ge$: C 52.39, H 7.69, N 7.64; found: C 52.18, H 7.97, N 7.42; IR (cm^{-1}): 2994 (w), 2970 (w), 2931 (w), 2904 (w), 2968 (w), 2579 (m), 2547 (w), 2489 (w), 1578 (w), 1516 (w), 1472 (w), 1446 (m), 1393 (s), 1365 (m), 1256 (m), 1227 (w), 1197 (m), 1159 (w), 1083 (w), 1069 (m), 1034 (w), 1023 (w), 983 (w), 928 (w), 880 (w), 840 (w), 788 (m), 756 (s), 731 (m), 706 (m), 679 (w), 628 (m), 615 (m), 580 (w), 567 (w).

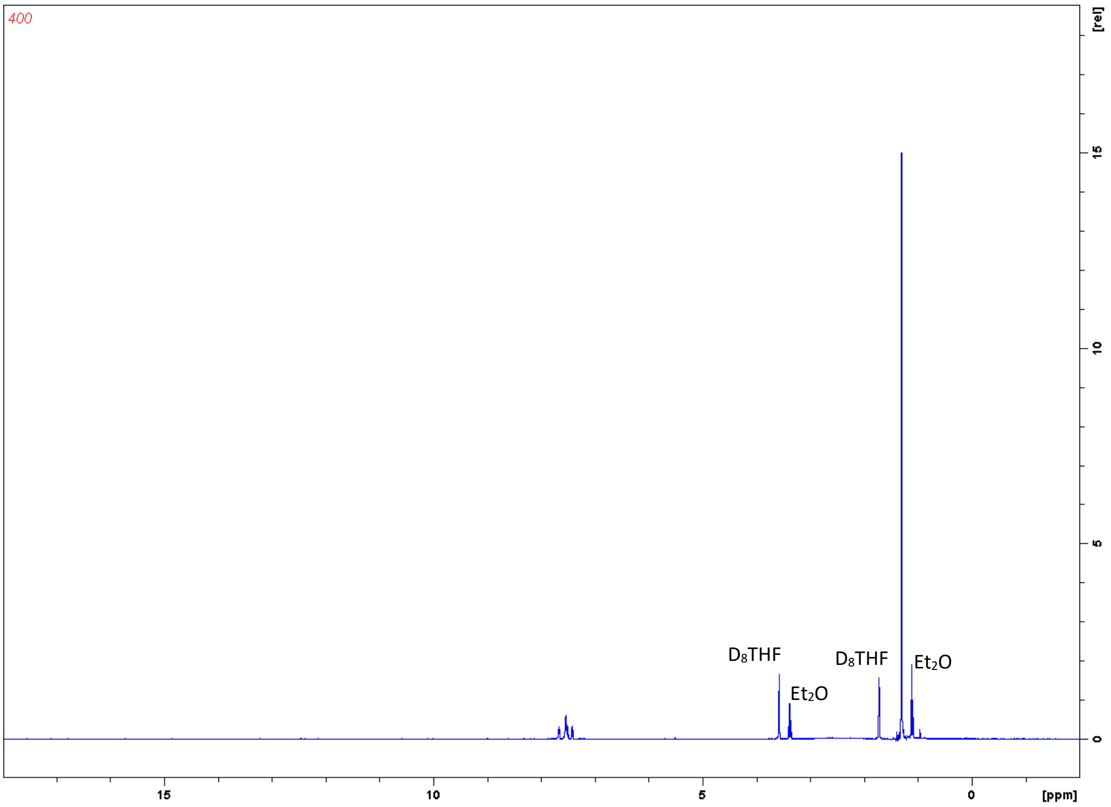

Figure S1. ^{1}H NMR spectrum of compound **3** (400 MHz, D_8-THF, 298K).

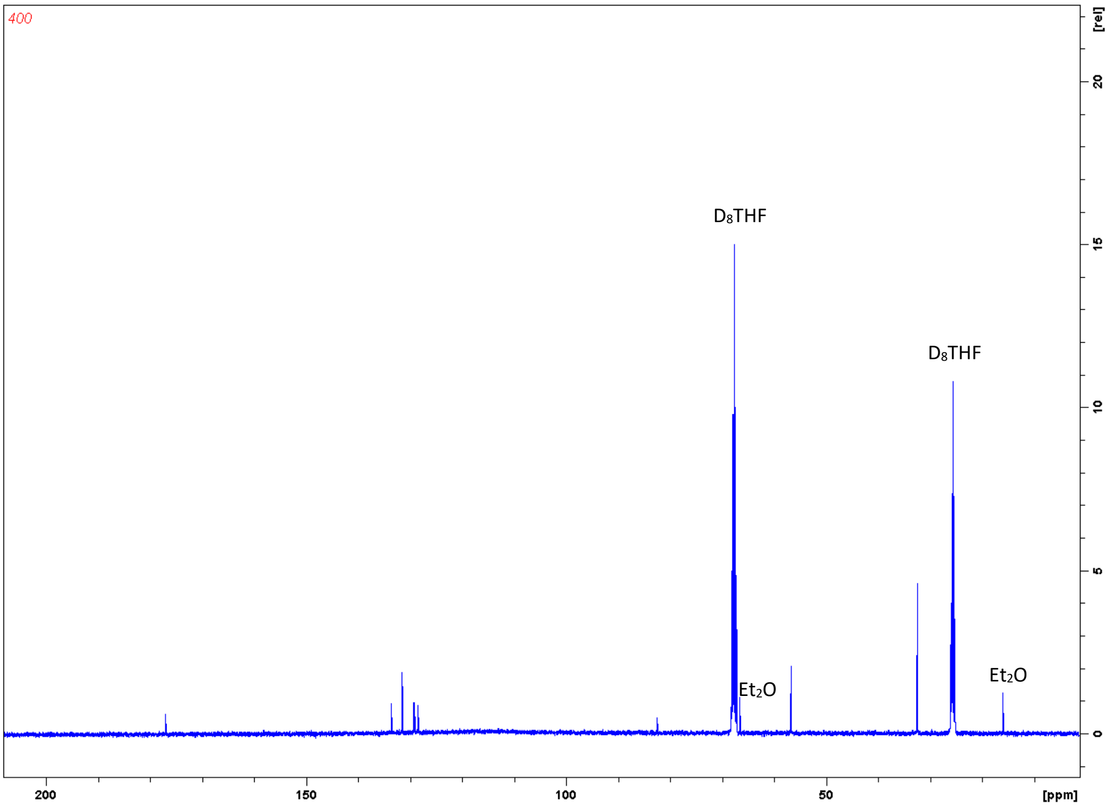

Figure S2. $^{13}C\{^{1}H\}$ NMR spectrum of compound **3** (100 MHz, D_8-THF, 298K).

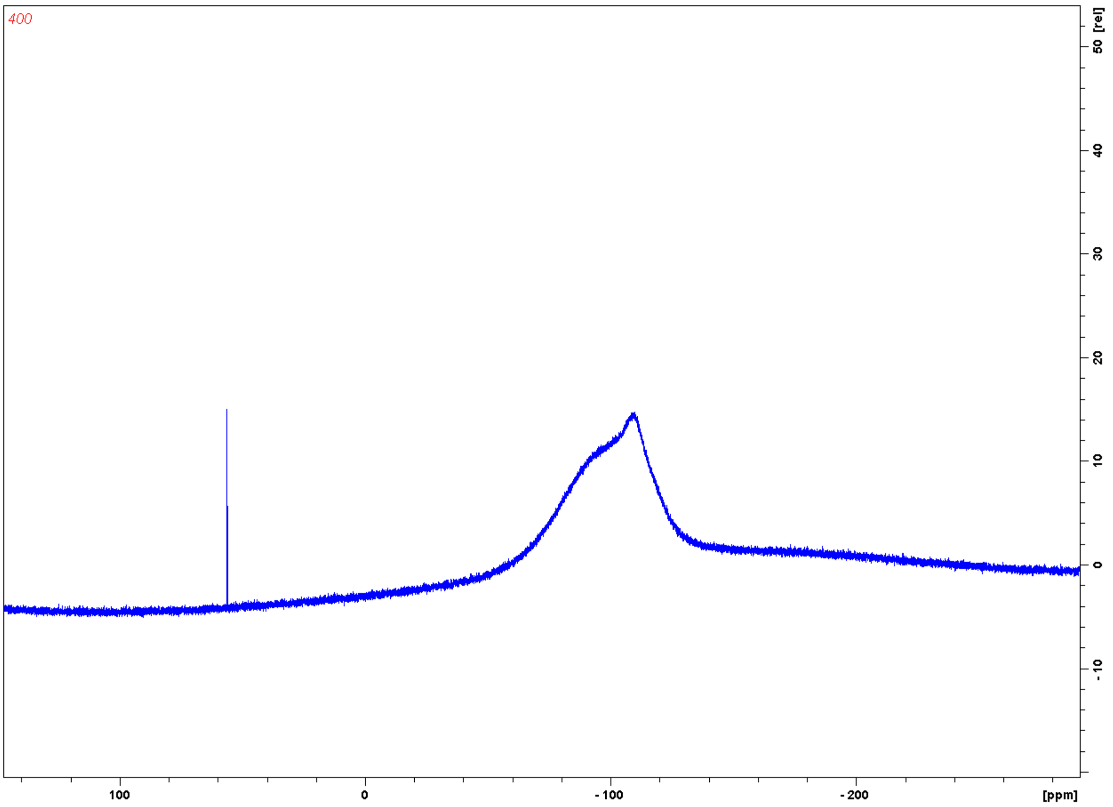

Figure S3. $^{29}Si\{^{1}H\}$ NMR spectrum of compound **3** (99.49 MHz, D_8-THF, 298 K).

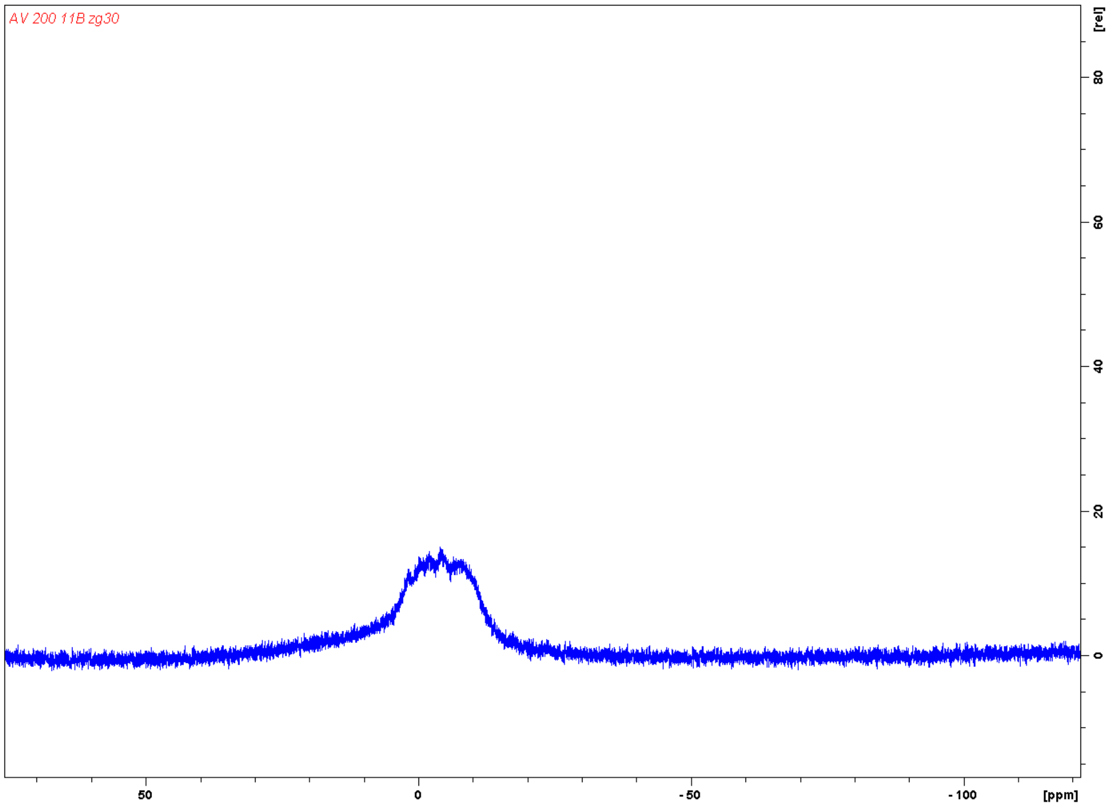

Figure S4. $^{11}B\{^{1}H\}$ NMR spectrum of compound **3** (64 MHz, D_8-THF, 298 K).

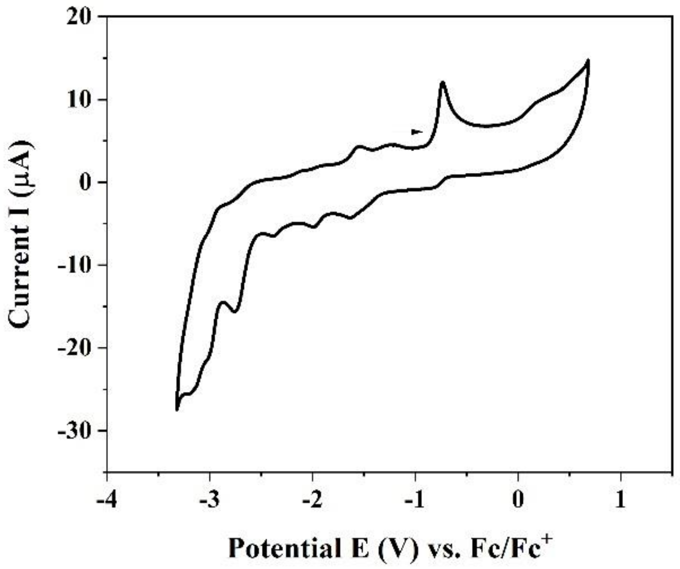

Figure S5. CV of compound **3** (1 mM in THF/ 0.3 M $TBAPF_6$) at a scan rate of v = 100 mV s^{-1}. No reversible redox event occurred on the explored range of potential. Several irreversible reduction peaks (E_{pc} = -1.63; -2.00; -2.39; -2.76 V) and irreversible oxidation (E_{pa} = 0.19 V) are observed.

Synthesis of 4

To a cooled (-20 °C) solution of **3** (0.16 g, 0.22 mmol) in 6 mL THF was added dropwise a potassium naphthalenide THF solution prepared by stirring 8.5 mg potassium (0.22 mmol) and 28.0 mg naphthalene (0.22 mmol) in 6 mL THF at room temperature from 2 hours. The reaction solution was allowed to warm to room temperature and a crystalline precipitate formed gradually. After stirring for 1 h, volatiles were removed under vacuum and the residue was washed with Et_2O (2 × 10 mL) to afford compound **4** as a dark red crystalline solid (0.19 g, 95 % isolated yield). Single crystals of **4** suitable for X-ray diffraction analysis were obtained from a resulting reaction solution without stirring at room temperature. M.p. 234 ºC (decomp.). Elemental analysis calcd for $C_{80}H_{144}N_8B_{20}Si_4Ge_2K_2O_4$: C 52.39, H 7.91, N 6.11; found: C 51.91, H 7.72, N 6.28; IR (cm^{-1}): 2966 (w), 2929 (w), 2903 (w), 2868 (w), 2457 (m), 2430 (m), 1517 (w), 1469 (w), 1446 (w), 1413 (s), 1394 (w), 1362 (m), 1267 (m), 1202 (s), 1146 (s), 1079 (w), 1054 (m), 1023 (w), 929 (w), 912 (w), 894 (w), 796 (m), 753 (s), 708 (s), 658 (w), 629 (m), 615 (m).

Synthesis of 5

To a brown-red solution of **3** (0.22 g, 0.30 mmol) in 10 mL THF was added a solution of $[Cp_2Fe][B\{C_6H_3(CF_3)_2\}_4]$ (0.32 g, 0.30 mmol) in 10 mL THF at room temperature with stirring. After addition, the reaction mixture was stirred further for 1 h and the color of the solution changed to orange. Volatiles were removed under vacuum and the residue was washed with Et_2O (3 × 10 mL) to afford compound **5** as an orange powder after dried under vacuum (0.44 g, 92 % isolated yield). Single crystals of **5** suitable for X-ray diffraction analysis were obtained from a concentrated THF solution of **5** by adding diethyl ether at room temperature. M.p. 121 °C (decomp.). ^{1}H NMR (200 MHz, D_8-THF, 298K): δ = 1.46 (s, 36 H, NC(C*H*$_3$)$_3$), 2.3 – 3.2 (vb, 10 BH, very broad and unresolved), 7.42-7.49 (m, 2 H, Ph-*H*), 7.53-7.65 (m, 8 H, Ph-*H*), 7.70-7.80 ppm (m, 12 H, Ph-*H*). ^{13}C{^{1}H} NMR (50 MHz, D_8-THF, 298K): δ = 32.7 (s, NC(*C*H$_3$)$_3$), 59.1 (s, N*C*(CH$_3$)$_3$), 74.6 (s, carborane-C), 118.0-118.5 (m, Barf-Ph-*C*), 125.6 (q, $J_{C\text{-}F}$ = 272 Hz, Barf-*C*F$_3$), 126.7 (Ph-*C*), 128.9-131.2 (qm, $J_{C\text{-}F}$ = 24 Hz, Barf-Ph-*C*), 129.4, 130.0 130.4, 130.8, 134.1 (s, Ph-*C*), 135.7 (m, Barf-Ph-*C*), 162.9 (q, $J_{C\text{-}B}$ = 37 Hz, Barf-Ph-*C*), 185.1 ppm (s, N*C*N). ^{29}Si{^{1}H} NMR (99.49 MHz, D_8-THF, 298 K): δ = 68.0 ppm (vb, *Si*$_2$Ge). ^{11}B{^{1}H} NMR (64 MHz, D_8-THF, 298 K): δ = -25 - 15 (m, vb, Carborane), -6.6 ppm (s, Barf-*B*); ^{19}F NMR (188 MHz, D_8-THF, 298 K): δ = -63.0 ppm (Barf-C*F*$_3$). Elemental analysis calcd for $C_{128}H_{136}B_{22}N_8Si_4Ge_2F_{48}$: C 48.14, H 4.29, N 3.51; found: C 47.86, H 4.08, N 3.29; IR (cm^{-1}): 2976 (w), 2937 (w), 2913 (w), 2874 (w), 2583 (m), 1610 (w), 1513 (w), 1474 (w), 1449 (w), 1401 (w), 1380 (m), 1370 (m), 1353 (s), 1273 (s), 1180 (w), 1160 (w), 1117 (s), 1094 (m), 1073 (w), 1023 (w), 1001 (w), 929 (w), 886 (m), 839 (m), 790 (w), 770 (m), 760 (w), 738 (w), 712 (m), 705 (m), 681 (s), 669 (m), 624 (w).

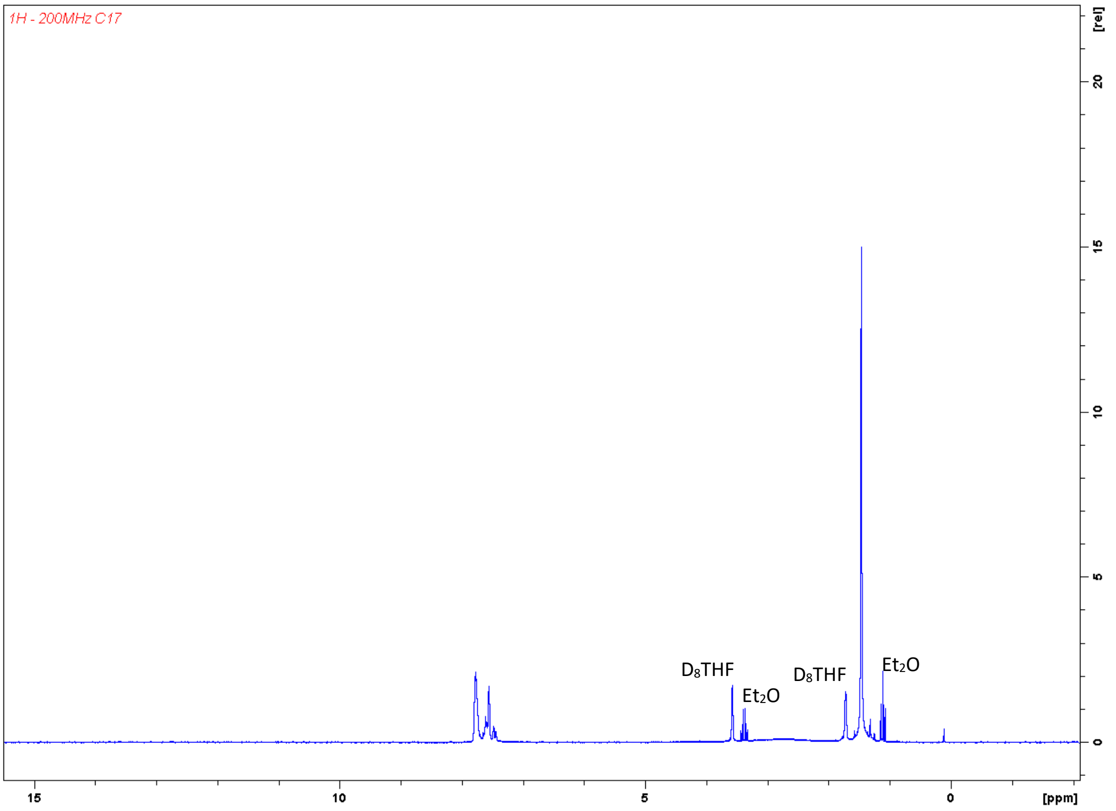

Figure S6. ^{1}H NMR spectrum of compound **5** (200 MHz, D_8-THF, 298K).

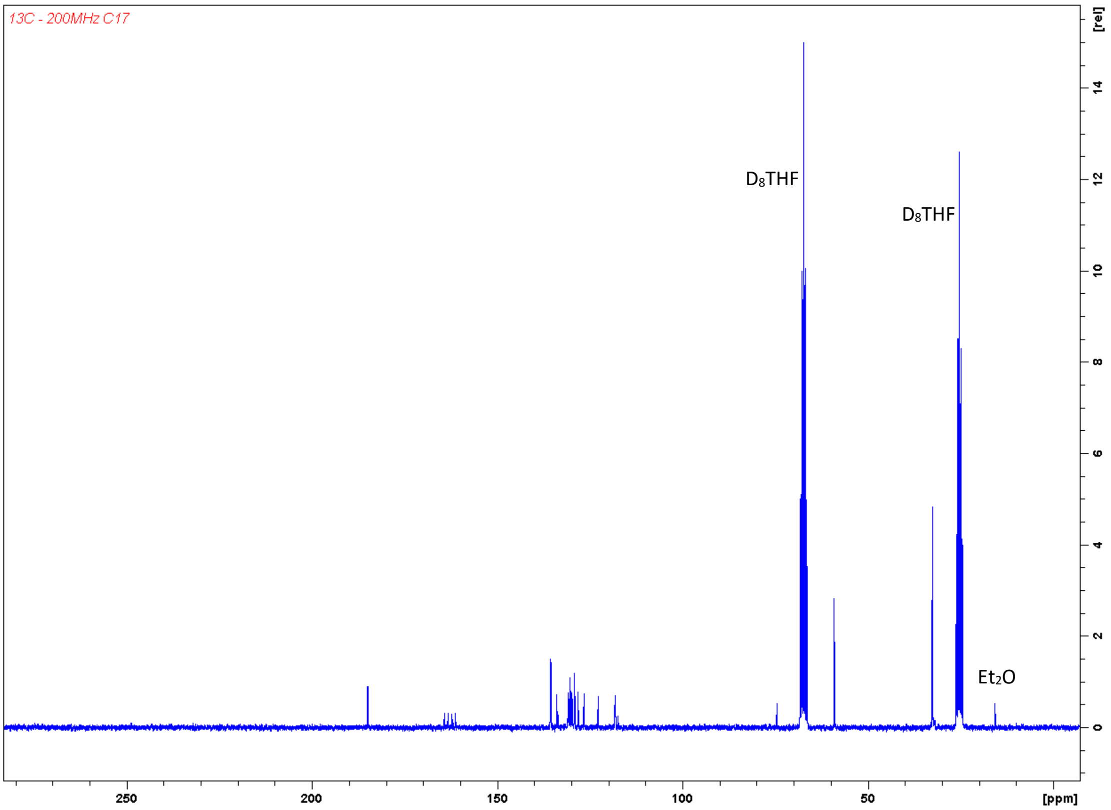

Figure S7. $^{13}C\{^{1}H\}$ NMR spectrum of compound **5** (50 MHz, D_8-THF, 298K).

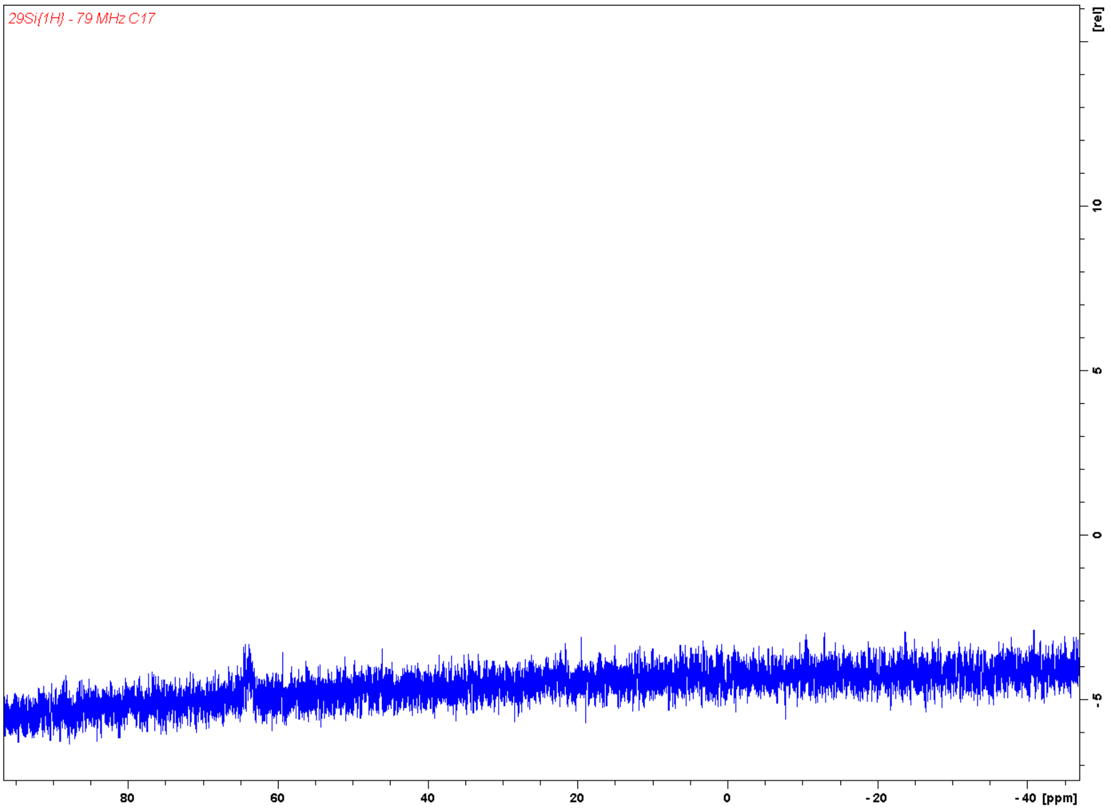

Figure S8. $^{29}Si\{^{1}H\}$ NMR spectrum of compound **5** (99 MHz, D_8-THF, 298 K).

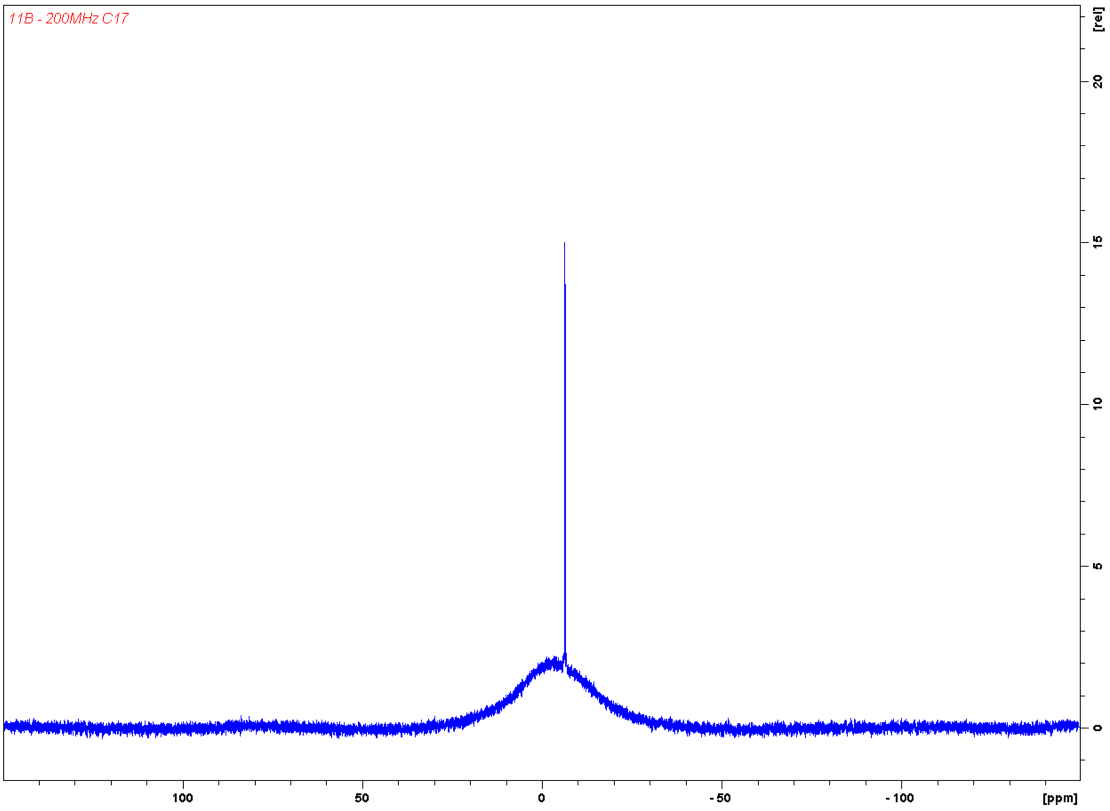

Figure S9. $^{11}B\{^{1}H\}$ NMR spectrum of compound **5** (64 MHz, D_8-THF, 298 K).

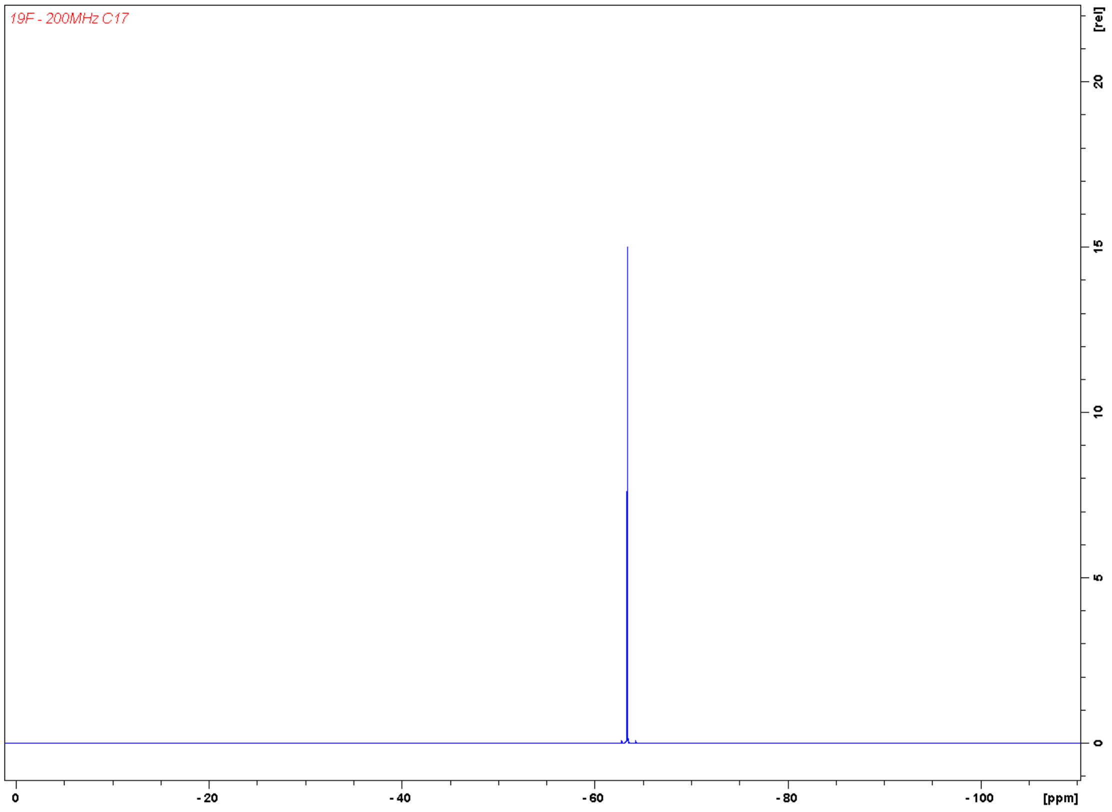

Figure S10. ^{19}F NMR spectrum of compound **5** (94 MHz, D_8-THF, 298 K).

Synthesis of 6

To a solution of **3** (0.21 g, 0.28 mmol) in 8 mL THF was added dropwise a solution of $GeCl_2$-dioxane (0.033 g, 0.14 mmol) in 8 mL THF at room temperature. A yellow precipitate formed immediately. After stirring for 1 h, volatiles were removed under reduced pressure and the residue was washed with Et_2O (2 × 10 mL) to afford compound **6** as a yellow powder after dried under vacuum (0.22 g, 95 % isolated yield). Single crystals of **6** suitable for X-ray diffraction analysis were obtained from a concentrated THF solution of **6** at room temperature. M.p. 272 °C (decomp.). Solid state ^{29}Si CP/MAS NMR (298 K): δ = 56.0 ppm. **Elemental analysis** calcd for $C_{64}H_{112}B_{20}N_8Si_4Cl_2Ge_3$: C 47.72, H 7.01, N 6.96; found: C 47.36, H 7.33, N 6.78; **IR** (cm^{-1}): 2993 (w), 2971 (w), 2929 (w), 2905 (w), 2865 (w), 2603 (m), 2563 (m), 1517 (w), 1472 (m), 1444 (m), 1397 (s), 1365 (s), 1261 (m), 1223 (w), 1195 (s), 1086 (w), 1067 (m), 1034 (w), 1026 (w), 969 (w), 931 (w), 908 (w), 883 (w), 837 (m), 792 (m), 760 (s), 736 (w), 726 (m), 709 (s), 680 (w), 671 (w), 635 (s), 625 (w).

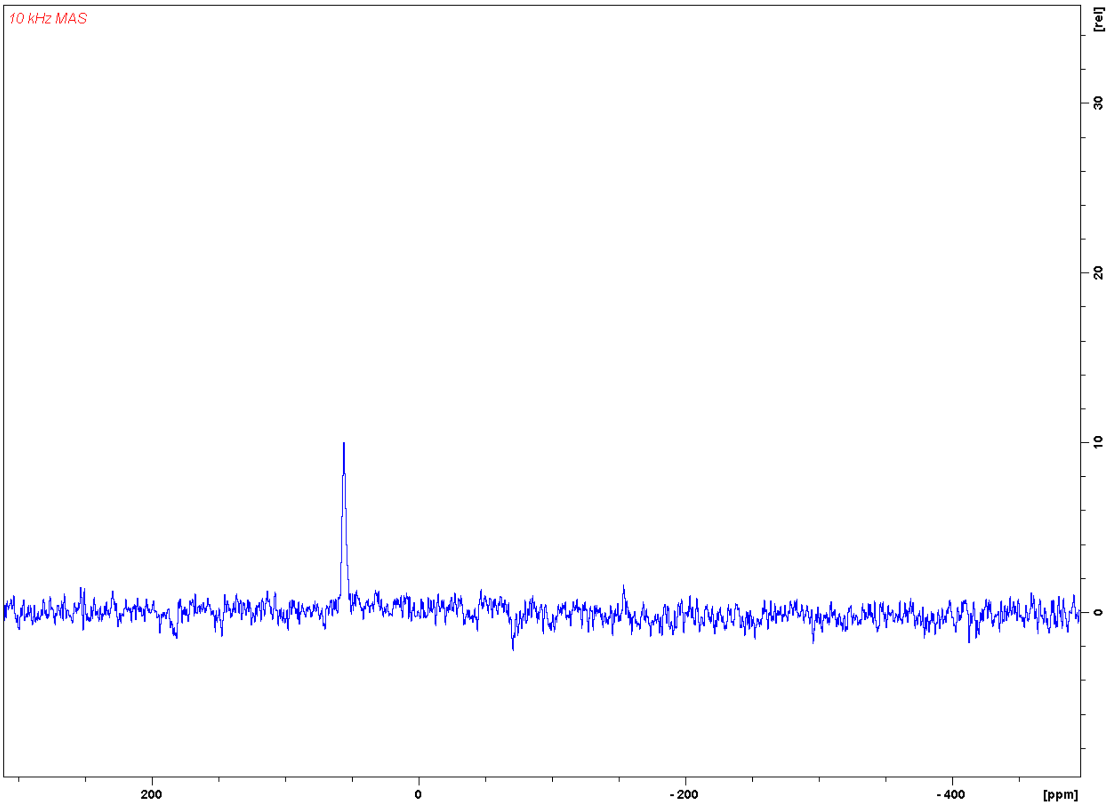

Figure S11. Solid state ^{29}Si CP/MAS NMR spectrum of compound **6** (298 K).

Synthesis of 7

To a cooled (-20 °C) suspension of **6** (0.32 g, 0.20 mmol) in 12 mL THF was added dropwise a potassium naphthalenide THF solution prepared by stirring 15.5 mg potassium (0.40 mmol) and 50.9 mg naphthalene (0.40 mmol) in 12 mL THF at room temperature for 2 hours. The reaction solution was allowed to warm to room temperature and stirred further for 1 h. Volatiles were removed under vacuum and the residue was washed with n-hexane (2 × 10 mL) and extracted with Et_2O (2 × 10 mL) as well as 5 mL THF. Concentration of the diethyl ether solution afforded **3** as brown-red crystals (0. 16 g, 53 %) at -30°C in 24 hours, while concentration of the THF solution allowed crystallization of **7** as dark-red crystals at 4 °C in 3 days (0.082 g, 28 % isolated yield). M.p. 204 ºC (decomp.). **Elemental analysis** calcd for $C_{64}H_{112}N_8B_{20}Si_4Ge_2$: C 52.39, H 7.69, N 7.64; found: C 51.96, H 7.52, N 7.45; **IR** (cm^{-1}): 2970 (w), 2932 (w), 2906 (w), 2869 (w), 2549 (w), 2478 (m), 1516 (w), 1472 (w), 1445 (w), 1394 (s), 1364 (m), 1263 (w), 1196 (m), 1084 (w), 1069 (w), 1042 (w), 1023 (w), 927 (w), 886 (w), 840 (w), 795 (w), 758 (s), 726 (m), 707 (s), 627 (m), 615 (m).

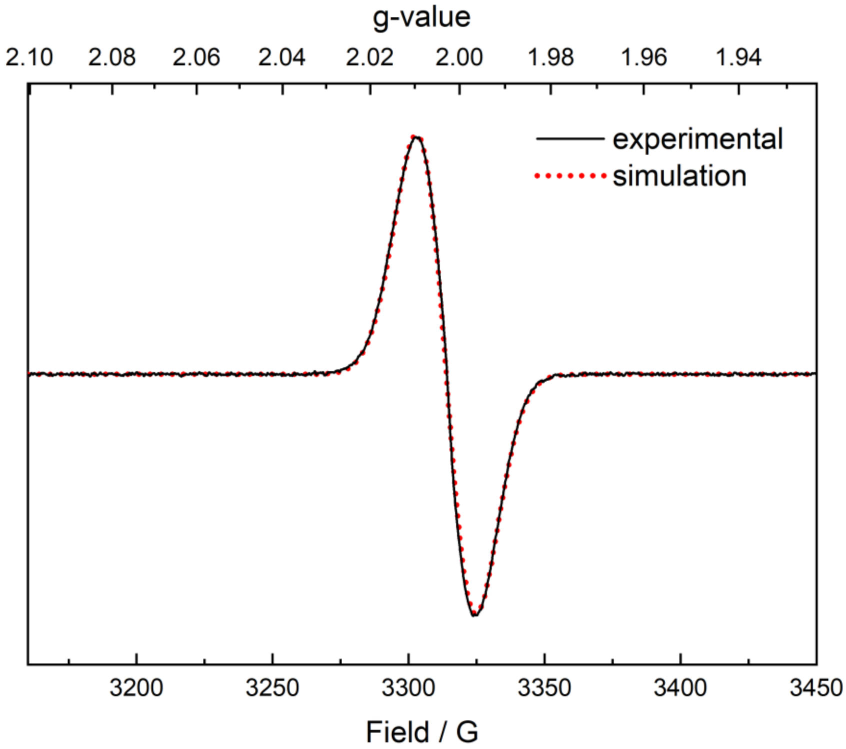

Figure S12. EPR spectrum of compound **7** in THF at 293 K. The g-value is 2.004and the line width 21.9 G. The spectrum was accumulated with a modulation amplitude of 1 G, 1 mW microwave power and 9.3 GHz microwave frequency.

A4. Crystallographic data

Table S1. Crystal data and structure refinement for **3**.

Empirical formula	C40 H76 B10 Ge N4 O2 Si2	
Formula weight	881.91	
Temperature	150(2) K	
Wavelength	1.54184 Å	
Crystal system	Triclinic	
Space group	P-1	
Unit cell dimensions	a = 13.6652(6) Å	α= 66.754(4)°.
	b = 13.8715(6) Å	β= 78.749(4)°.
	c = 16.3415(8) Å	γ = 62.009(4)°.
Volume	2513.0(2) $Å^3$	
Z	2	
Density (calculated)	1.165 Mg/m^3	
Absorption coefficient	1.551 mm^{-1}	
F(000)	940	
Crystal size	0.350 x 0.190 x 0.120 mm^3	
Theta range for data collection	2.943 to 67.500°.	
Index ranges	-16<=h<=16, -16<=k<=16, -19<=l<=17	
Reflections collected	17252	
Independent reflections	9049 [R(int) = 0.0332]	
Completeness to theta = 67.500°	99.8 %	
Absorption correction	Semi-empirical from equivalents	
Max. and min. transmission	1.00000 and 0.72305	
Refinement method	Full-matrix least-squares on F^2	
Data / restraints / parameters	9049 / 0 / 548	
Goodness-of-fit on F^2	1.033	
Final R indices [I>2sigma(I)]	R1 = 0.0355, wR2 = 0.0884	
R indices (all data)	R1 = 0.0427, wR2 = 0.0943	
Extinction coefficient	n/a	
Largest diff. peak and hole	0.413 and -0.482 e.$Å^{-3}$	

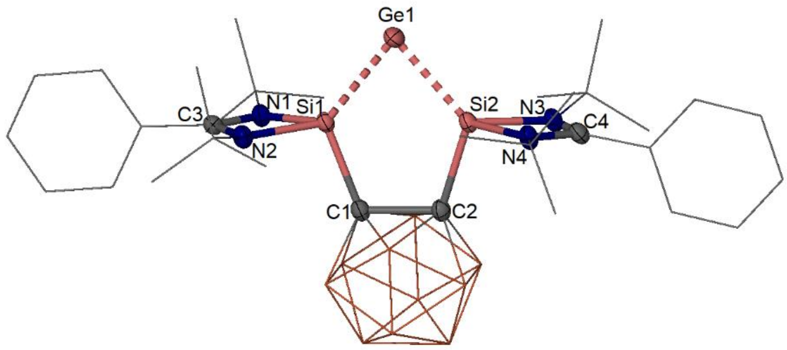

Figure S13. Molecular structure of compound **3**. Thermal ellipsoids are drawn at 50% probability level. H atoms are omitted for clarity.

Table S2. Selected interatomic distances [Å] and angles [°] for compound **3**.

Bond distances		Bond angels	
Ge1-Si2	2.2846(5)	Si2-Ge1-Si1	80.59(2)
Ge1-Si1	2.2896(5)	N3-Si2-N4	70.50(6)
Si2-N3	1.861(2)	N3-Si2-C2	100.44(7)
Si2-N4	1.867(1)	N4-Si2-C2	103.54(7)
Si2-C2	1.946(2)	N3-Si2-Ge1	126.24(5)
Si1-N1	1.863(1)	N4-Si2-Ge1	124.27(5)
Si1-N2	1.864(1)	C2-Si2-Ge1	120.30(5)
Si1-C1	1.937(2)	N1-Si1-N2	70.59(6)
N4-C4	1.340(2)	N1-Si1-C1	103.45(7)
N3-C4	1.335(2)	N2-Si1-C1	103.64(7)
N1-C3	1.344(2)	N1-Si1-Ge1	123.43(5)
N2-C3	1.336(2)	N2-Si1-Ge1	124.32(5)
C2-C1	1.671(2)	C1-Si1-Ge1	120.24(5)
		C4-N4-Si2	91.0(1)
		C4-N3-Si2	91.4(1)
		C3-N1-Si1	91.0(1)
		C3-N2-Si1	91.2(1)

	C1-C2-Si2	109.1(1)
	C2-C1-Si1	109.6(1)
	N3-C4-N4	107.1(2)
	N2-C3-N1	107.0(2)

Table S3. Crystal data and structure **4**

Empirical formula	C80 H144 B20 Ge2 K2 N8 O4 Si4	
Formula weight	1833.96	
Temperature	150(2) K	
Wavelength	1.54184 Å	
Crystal system	Monoclinic	
Space group	C2/c	
Unit cell dimensions	a = 20.7213(4) Å	α= 90°.
	b = 20.8524(3) Å	β= 104.553(2)°.
	c = 26.4574(5) Å	γ = 90°.
Volume	11065.2(4) $Å^3$	
Z	4	
Density (calculated)	1.101 Mg/m^3	
Absorption coefficient	2.089 mm^{-1}	
F(000)	3880	
Crystal size	0.230 x 0.110 x 0.040 mm^3	
Theta range for data collection	3.227 to 67.499°.	
Index ranges	-24<=h<=24, -23<=k<=24, -31<=l<=29	
Reflections collected	36156	
Independent reflections	9975 [R(int) = 0.0571]	
Completeness to theta = 67.499°	99.9 %	
Absorption correction	Semi-empirical from equivalents	
Max. and min. transmission	1.00000 and 0.56601	
Refinement method	Full-matrix least-squares on F^2	
Data / restraints / parameters	9975 / 432 / 700	
Goodness-of-fit on F^2	0.997	
Final R indices [I>2sigma(I)]	R1 = 0.0464, wR2 = 0.1135	
R indices (all data)	R1 = 0.0732, wR2 = 0.1277	
Extinction coefficient	n/a	
Largest diff. peak and hole	0.835 and -0.548 e.$Å^{-3}$	

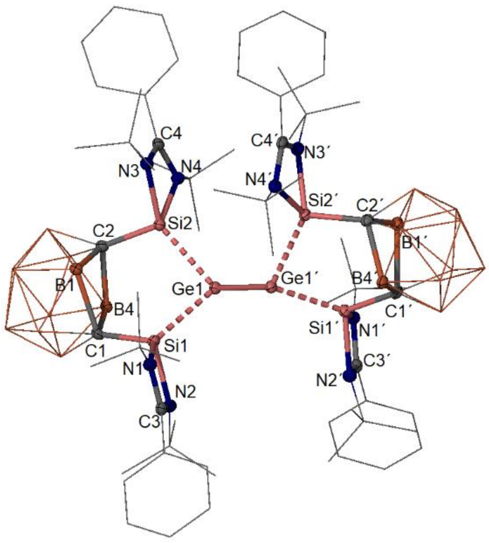

Figure S14. Molecular structure of the dianion of compound 4 (The carborane moieties and one of the *tert*-butyl groups are disordered over two orientations with an occupancy ratio of 0.50:0.50. Only one set of them is depicted.) Thermal ellipsoids are drawn at 30% probability level. H atoms are omitted for clarity. Symmetry transformations used to generate equivalent atoms with (´): -x,y,-z+3/2.

Table S4. Selected interatomic distances [Å] and angles [°] for compound 4.

Bond distances		Bond angels	
Ge1-Si2	2.4105(8)	Si2-Ge1-Si1	93.75(3)
Ge1-Si1	2.4294(8)	Si2-Ge1-Ge1´	123.56(2)
Ge1-Ge1´	2.5161(6)	Si1-Ge1-Ge1´	99.94(3)
Si2-C2	1.78(3)	C2-Si2-N4	108.8(9)
Si2-N4	1.861(2)	C2-Si2-N3	103.3(9)
Si2-N3	1.877(2)	N4-Si2-N3	70.1(1)
Si2-C4	2.306(3)	C2-Si2-C4	105.5(9)
Si1-C1	1.74(2)	C2-Si2-Ge1	112.7(9)
Si1-N1	1.862(3)	N4-Si2-Ge1	118.28(8)
Si1-N2	1.868(2)	N3-Si2-Ge1	135.58(8)
Si1-C3	2.310(3)	C4-Si2-Ge1	140.55(7)
N4-C4	1.333(4)	C1-Si1-N1	112.4(6)
N3-C4	1.331(4)	C1-Si1-N2	109.3(6)

N2-C3	1.326(4)	N1-Si1-N2	70.6(1)
N1-C3	1.345(4)	C1-Si1-C3	114.5(6)
C2-B1	1.65(3)	C1-Si1-Ge1	114.9(6)
C2-B4	1.79(2)	N1-Si1-Ge1	125.44(8)
		N2-Si1-Ge1	115.57(9)
		C4-N4-Si2	90.9(2)
		C4-N3-Si2	90.3(2)
		C3-N2-Si1	91.0(2)
		C3-N1-Si1	90.7(2)
		B1-C2-Si2	109.1(2)
		B1-C2-B4	86.0(1)
		Si2-C2-B4	110.1(2)
		N3-C4-N4	107.4(2)
		N2-C3-N1	107.6(3)

Table S5. Crystal data and structure **5**

Empirical formula	C140 H166 B22 F48 Ge2 N8 O3 Si4	
Formula weight	3416.16	
Temperature	150(2) K	
Wavelength	1.54184 Å	
Crystal system	Triclinic	
Space group	P-1	
Unit cell dimensions	a = 16.4458(4) Å	α= 75.181(2)°.
	b = 17.5227(4) Å	β= 77.455(2)°.
	c = 32.2902(7) Å	γ = 66.465(2)°.
Volume	8177.6(4) $Å^3$	
Z	2	
Density (calculated)	1.387 Mg/m^3	
Absorption coefficient	1.665 mm^{-1}	
F(000)	3496	
Crystal size	0.340 x 0.230 x 0.040 mm^3	
Theta range for data collection	2.801 to 67.498°.	
Index ranges	-19<=h<=19, -20<=k<=19, -37<=l<=38	
Reflections collected	57529	
Independent reflections	29415 [R(int) = 0.0301]	
Completeness to theta = 67.498°	99.9 %	
Absorption correction	Semi-empirical from equivalents	
Max. and min. transmission	1.00000 and 0.62231	
Refinement method	Full-matrix least-squares on F^2	
Data / restraints / parameters	29415 / 18 / 2214	
Goodness-of-fit on F^2	1.026	
Final R indices [I>2sigma(I)]	R1 = 0.0447, wR2 = 0.1154	
R indices (all data)	R1 = 0.0572, wR2 = 0.1258	
Extinction coefficient	n/a	
Largest diff. peak and hole	0.911 and -0.617 e.$Å^{-3}$	

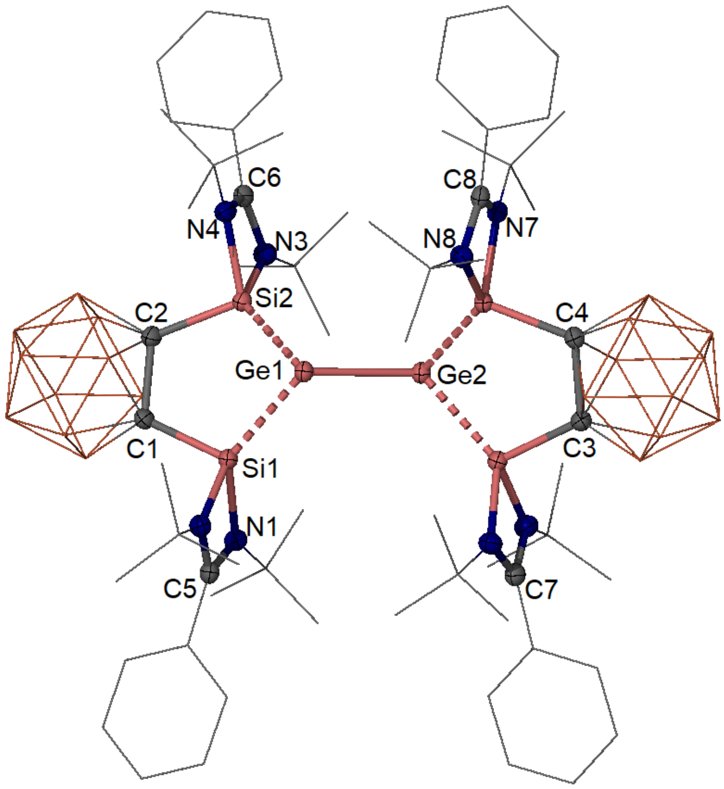

Figure S15. Molecular structure of the dication of compound **5**. Thermal ellipsoids are drawn at 30% probability level. H atoms are omitted for clarity.

Table S6. Selected interatomic distances [Å] and angles [°] for compound **5**.

Bond distances		Bond angels	
Ge1-Si1	2.3934(6)	Si1-Ge1-Si2	84.05(2)
Ge1-Si2	2.4044(6)	Si1-Ge1-Ge2	114.85(2)
Ge1-Ge2	2.5468(3)	Si2-Ge1-Ge2	100.64(2)
Ge2-Si3	2.3967(6)	Si3-Ge2-Si4	84.52(2)
Ge2-Si4	2.4015(6)	Si3-Ge2-Ge1	116.22(2)
Si1-N1	1.815(2)	Si4-Ge2-Ge1	100.19(2)
Si1-N2	1.831(2)	N1-Si1-Ge1	136.54(7)
Si2-N4	1.818(2)	N2-Si1-Ge1	119.82(7)
Si2-N3	1.822(2)	C1-Si1-Ge1	109.84(7)
Si3-N6	1.816(2)	C5-Si1-Ge1	140.85(6)
Si3-N5	1.838(2)	N4-Si2-Ge1	122.02(6)
Si4-N8	1.823(2)	N3-Si2-Ge1	134.34(6)
Si4-N7	1.823(2)	C2-Si2-Ge1	109.82(7)

Si1-C1	1.929(2)	C6-Si2-Ge1	141.51(6)
Si2-C2	1.924(2)	N6-Si3-Ge2	136.79(7)
Si3-C3	1.922(2)	N5-Si3-Ge2	120.21(7)
Si4-C4	1.924(2)	C3-Si3-Ge2	109.44(7)
N1-C5	1.345(3)	C7-Si3-Ge2	140.15(6)
N2-C5	1.334(3)	N8-Si4-Ge2	134.70(6)
N3-C6	1.345(3)	N7-Si4-Ge2	121.59(6)
N4-C6	1.344(3)	C4-Si4-Ge2	109.47(7)
N5-C7	1.334(3)	C8-Si4-Ge2	140.87(6)
N6-C7	1.345(3)	N1-Si1-N2	72.42(9)
N7-C8	1.340(3)	N1-Si1-C1	106.00(9)
N8-C8	1.349(3)	N2-Si1-C1	104.25(9)
C1-C2	1.673(3)	C1-Si1-C5	107.16(9)
C3-C4	1.683(3)	N4-Si2-N3	72.68(9)
		N4-Si2-C2	101.94(9)
		N3-Si2-C2	107.93(9)
		C2-Si2-C6	106.81(8)
		N6-Si3-N5	72.27(9)
		N6-Si3-C3	106.43(9)
		N5-Si3-C3	103.55(9)
		C3-Si3-C7	108.14(8)
		N8-Si4-N7	72.40(8)
		N8-Si4-C4	107.74(9)
		N7-Si4-C4	103.25(9)
		C4-Si4-C8	107.87(8)
		C5-N1-Si1	90.4(1)
		C2-C1-Si1	113.7(1)
		C5-N2-Si1	90.0(1)
		C1-C2-Si2	113.4(1)
		C6-N3-Si2	90.1(1)
		C4-C3-Si3	114.2(1)
		C6-N4-Si2	90.3(1)
		C3-C4-Si4	113.2(1)
		C7-N5-Si3	90.1(1)
		C9-C5-Si1	171.3(2)
		C7-N6-Si3	90.6(1)
		C8-N7-Si4	90.6(1)
		C8-N8-Si4	90.4(1)

	N2-C5-N1	107.0(2)
	N4-C6-N3	106.7(2)
	N5-C7-N6	107.1(2)
	N7-C8-N8	106.4(2)

Table S7. Crystal data and structure **6**

Empirical formula	C64 H112 B20 Cl2 Ge3 N8 Si4	
Formula weight	1610.84	
Temperature	150(2) K	
Wavelength	0.71073 Å	
Crystal system	Triclinic	
Space group	P1	
Unit cell dimensions	a = 14.3158(11) Å	α= 73.401(5)°.
	b = 14.4661(10) Å	β= 86.882(5)°.
	c = 16.2213(9) Å	γ = 60.481(8)°.
Volume	2786.4(4) $Å^3$	
Z	1	
Density (calculated)	0.960 Mg/m^3	
Absorption coefficient	0.927 mm^{-1}	
F(000)	838	
Crystal size	0.150 x 0.110 x 0.060 mm^3	
Theta range for data collection	1.317 to 26.361°.	
Index ranges	-15<=h<=17, -17<=k<=18, -19<=l<=19	
Reflections collected	20396	
Independent reflections	13478 [R(int) = 0.0564]	
Completeness to theta = 25.242°	99.6 %	
Absorption correction	Semi-empirical from equivalents	
Max. and min. transmission	1.00000 and 0.36382	
Refinement method	Full-matrix least-squares on F^2	
Data / restraints / parameters	13478 / 807 / 1186	
Goodness-of-fit on F^2	0.926	
Final R indices [I>2sigma(I)]	R1 = 0.0686, wR2 = 0.1784	
R indices (all data)	R1 = 0.1085, wR2 = 0.2082	
Absolute structure parameter	0.468(17)	
Extinction coefficient	n/a	
Largest diff. peak and hole	0.502 and -0.384 e.$Å^{-3}$	

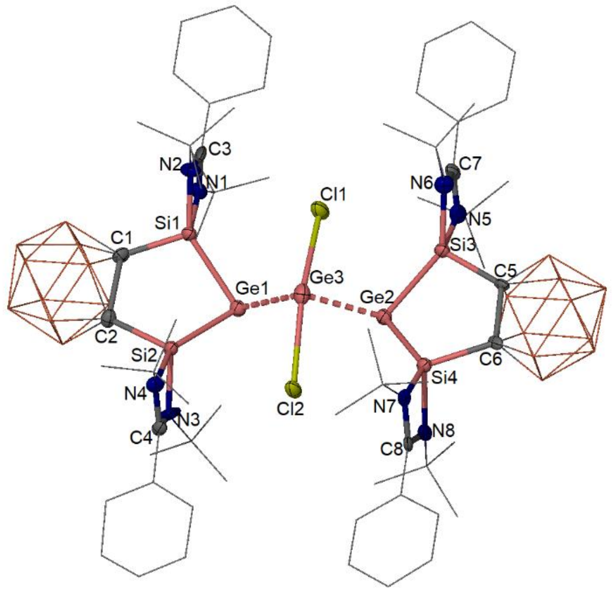

Figure S16. Molecular structure of compound 6. (The GeCl2 moiety is disordered over two orientations with an occupancy ratio of 0.50:0.50. Only one set is depicted.) Thermal ellipsoids are drawn at 30% probability level. H atoms are omitted for clarity.

Table S8. Selected interatomic distances [Å] and angles [°] for compound 6.

Bond distances		Bond angels	
Ge1-Ge3	2.412(4)	Si1-Ge1-Si2	86.9(2)
Ge2-Ge3	2.416(5)	Si1-Ge1-Ge3	103.3(2)
Ge1-Si1	2.363(4)	Si2-Ge1-Ge3	104.6(2)
Ge1-Si2	2.375(4)	Ge3-Ge2-Si3	106.5(2)
Ge2-Si3	2.435(5)	Ge3-Ge2-Si4	104.9(2)
Ge2-Si4	2.442(5)	Si3-Ge2-Si4	84.0(2)
Cl1-Ge3	2.556(5)	Ge1-Ge3-Ge2	110.3(2)
Cl2-Ge3	2.586(5)	Ge1-Ge3-Cl1	86.9(1)
Si1-N2	1.76(1)	Ge2-Ge3-Cl1	83.3(2)
Si1-N1	1.86(2)	Ge1-Ge3-Cl2	86.2(2)
Si2-N3	1.81(1)	Ge2-Ge3-Cl2	84.9(2)
Si2-N4	1.81(1)	Cl1-Ge3-Cl2	163.2(2)
Si3-N5	1.80(1)	N3-Si2-Ge1	109.3(4)
Si3-N6	1.82(1)	N4-Si2-Ge1	143.8(4)
Si4-N7	1.76(2)	C2-Si2-Ge1	106.6(4)
Si4-N8	1.84(1)	C4-Si2-Ge1	133.2(3)
Si1-C1	1.88(1)	N2-Si1-Ge1	142.8(4)

Si1-C3	2.18(2)	C1-Si1-Ge1	105.5(4)
Si2-C2	1.94(1)	N5-Si3-Ge2	107.9(4)
Si2-C4	2.27(1)	N6-Si3-Ge2	142.3(4)
Si3-C5	1.92(1)	C5-Si3-Ge2	105.7(4)
Si3-C7	2.25(1)	C7-Si3-Ge2	130.2(3)
Si4-C6	1.99(1)	N8-Si4-Ge2	108.6(4)
Si4-C8	2.26(2)	C6-Si4-Ge2	108.1(4)
C1-C2	1.78(2)	N2-Si1-N1	68.5(8)
C5-C6	1.66(2)	N2-Si1-C1	109.1(6)
N2-C3	1.09(3)	N1-Si1-C1	107(1)
N3-C4	1.32(1)	C1-Si1-C3	1145(1)
N4-C4	1.37(2)	C2-C1-Si1	116.8(7)
N5-C7	1.29(2)	N3-Si2-N4	72.6(5)
N6-C7	1.38(2)	N3-Si2-C2	106.2(6)
N7-C8	1.37(2)	N4-Si2-C2	107.3(5)
N8-C8	1.40(3)	C2-Si2-C4	112.6(5)
C3-N1	1.38(2)	C3-N2-Si1	97(1)
		C1-C2-Si2	108.8(7)
		N5-Si3-N6	72.8(5)
		N5-Si3-C5	109.2(5)
		N6-Si3-C5	109.4(5)
		C5-Si3-C7	116.7(5)
		C4-N3-Si2	91.5(8)
		N7-Si4-N8	75.5(8)
		N7-Si4-C6	111.3(8)
		N8-Si4-C6	107.6(5)
		C6-Si4-C8	116(1)
		C4-N4-Si2	89.8(7)
		C7-N5-Si3	92.1(8)
		C6-C5-Si3	118.4(7)
		C7-N6-Si3	88.2(8)
		C5-C6-Si4	110.0(8)
		C8-N7-Si4	92(1)
		C8-N8-Si4	87(1)
		N5-C7-N6	107(1)
		N7-C8-N8	106(2)
		N3-C4-N4	106(1)

Table S9. Crystal data and structure **7**

Empirical formula	C64 H112 B20 Ge2 N8 Si4	
Formula weight	1467.35	
Temperature	150(2) K	
Wavelength	1.54184 Å	
Crystal system	Triclinic	
Space group	P-1	
Unit cell dimensions	a = 15.7103(13) Å	α= 70.904(8)°.
	b = 22.218(2) Å	β= 71.014(8)°.
	c = 24.120(2) Å	γ = 76.361(7)°.
Volume	7443.8(13) $Å^3$	
Z	3	
Density (calculated)	0.982 Mg/m^3	
Absorption coefficient	1.463 mm^{-1}	
F(000)	2316	
Crystal size	0.070 x 0.050 x 0.030 mm^3	
Theta range for data collection	2.499 to 67.497°.	
Index ranges	-18<=h<=17, -26<=k<=26, -27<=l<=28	
Reflections collected	50461	
Independent reflections	26558 [R(int) = 0.1152]	
Completeness to theta = 67.497°	99.0 %	
Absorption correction	Semi-empirical from equivalents	
Max. and min. transmission	1.00000 and 0.55308	
Refinement method	Full-matrix least-squares on F^2	
Data / restraints / parameters	26558 / 1295 / 1372	
Goodness-of-fit on F^2	0.710	
Final R indices [I>2sigma(I)]	R1 = 0.0793, wR2 = 0.1775	
R indices (all data)	R1 = 0.2449, wR2 = 0.2381	
Extinction coefficient	n/a	
Largest diff. peak and hole	1.021 and -0.527 e.$Å^{-3}$	

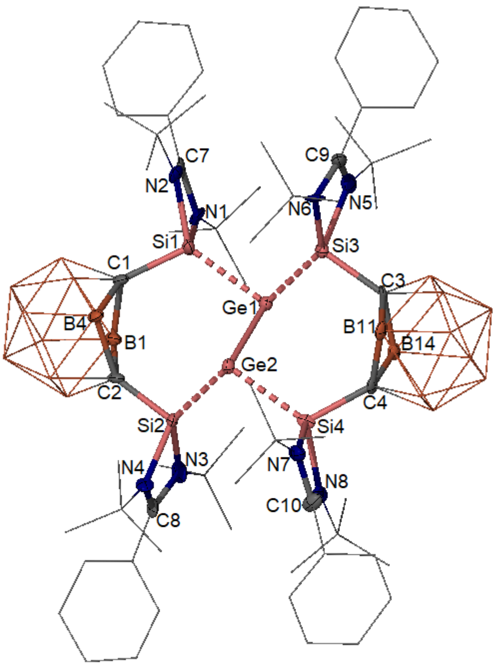

Figure S17. Molecular structure of compound **7**. (There are two independent molecules in the asymmetric unit, only one is depicted. In both structures, the two germanium atoms are disordered over two orientations with an occupancy ratio of 0.87:0.13). Thermal ellipsoids are drawn at 30% probability level. H atoms are omitted for clarity.

Table S10. Selected interatomic distances [Å] and angles [°] for compound **7**.

Bond distances		Bond angles	
Ge1-Ge2	2.559(2)	Si3-Ge1-Si1	106.5(1)
Ge1-Si3	2.384(3)	Si3-Ge1-Ge2	95.93(8)
Ge1-Si1	2.406(3)	Si1-Ge1-Ge2	103.10(8)
Ge2-Si2	2.406(3)	Si2-Ge2-Si4	107.3(1)
Ge2-Si4	2.411(3)	Si2-Ge2-Ge1	96.17(9)
Si3-N5	1.847(9)	Si4-Ge2-Ge1	103.70(8)
Si3-N6	1.891(8)	N5-Si3-Ge1	120.7(3)
Si4-N7	1.847(9)	N6-Si3-Ge1	139.3(3)
Si4-N8	1.829(8)	C3-Si3-Ge1	108.4(3)
Si2-N4	1.837(9)	C2-Si2-Ge2	107.3(3)
Si2-N3	1.838(8)	N4-Si2-Ge2	118.5(3)
Si1-N2	1.826(9)	N3-Si2-Ge2	139.5(3)
Si1-N1	1.843(9)	N8-Si4-Ge2	132.3(3)
Si3-C3	1.903(9)	C4-Si4-Ge2	116.0(3)
Si4-C4	1.836(9)	N7-Si4-Ge2	111.8(3)

Si2-C2	1.80(1)	N2-Si1-Ge1	133.1(3)
Si1-C1	1.84(1)	C1-Si1-Ge1	118.3(3)
N2-C7	1.36(1)	N1-Si1-Ge1	110.9(3)
N8-C10	1.32(1)	C7-N2-Si1	92.0(7)
N5-C9	1.33(1)	C10-N8-Si4	91.7(6)
N1-C7	1.33(1)	C9-N5-Si3	93.1(7)
N4-C8	1.34(1)	C7-N1-Si1	92.0(7)
N7-C10	1.37(1)	C8-N4-Si2	90.1(6)
N6-C9	1.34(1)	C10-N7-Si4	89.5(6)
N3-C8	1.34(1)	C9-N6-Si3	90.8(7)
		C8-N3-Si2	89.9(6)
		C2-Si2-N4	108.5(4)
		N5-Si3-N6	69.6(4)
		N5-Si3-C3	107.1(4)
		N6-Si3-C3	104.4(4)
		N8-Si4-C4	107.6(4)
		N8-Si4-N7	72.0(4)
		C4-Si4-N7	105.5(4)
		C2-Si2-N3	105.0(5)
		N4-Si2-N3	72.0(4)
		N2-Si1-C1	105.4(4)
		N2-Si1-N1	70.9(4)
		C1-Si1-N1	104.3(4)
		N5-C9-N6	106.4(9)
		N1-C7-N2	104.6(10)
		N4-C8-N3	107.4(9)
		N8-C10-N7	106.7(9)

B. Computational Section

All quantum chemical calculations were carried out using Gaussian 09.D01.[3] The geometries of all compounds were optimized at the B3PW91 [4]-D3 [5] level of theory with 6-311+G(d,p) basis set for Si and Ge and 6-31G(d,p) for all other atoms.[6] Analytical frequencies were computed to verify the stationary points. NBO analysis was carried out using the NBO 6.0 software.[7]

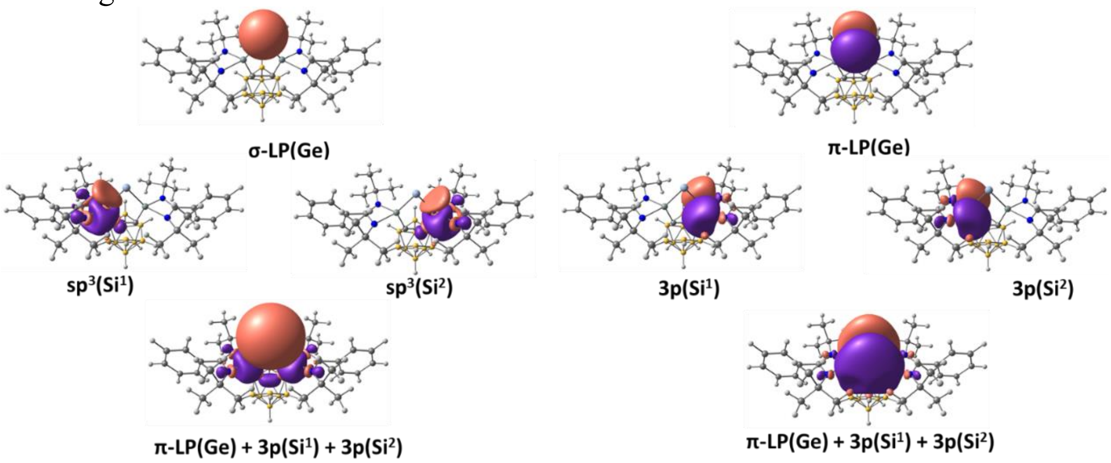

Figure S18. NBO analysis of composition of HOMO and HOMO-1 of **3**.

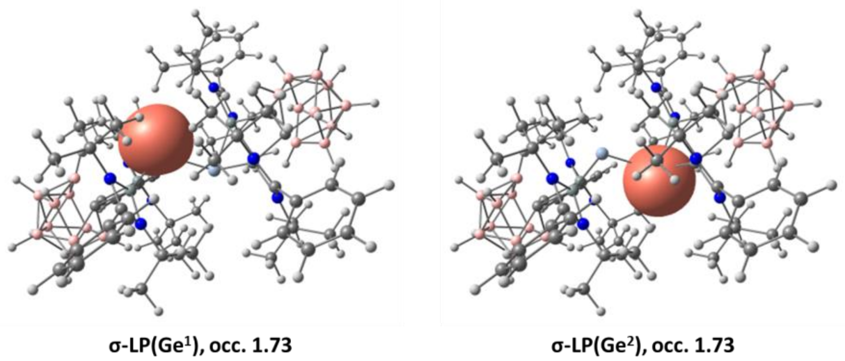

Figure S19. Selected NBOs of **5**.

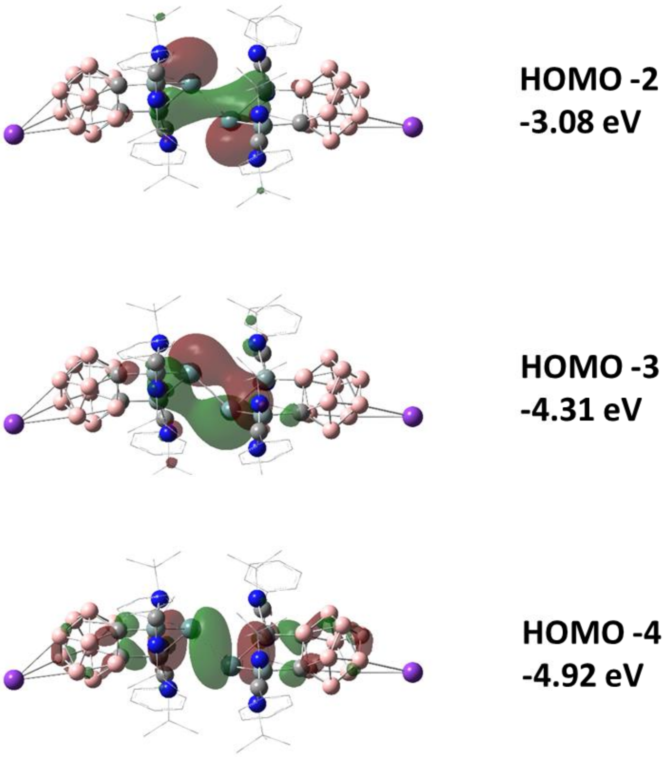

Figure S20. Selcted molecular orbitals of **4**. HOMO-4 represents the σ-bond between the Ge-Ge. HOMO-3, HOMO-2 correspond to the lone pairs of the cetral Ge-Ge. HOMO and HOMO-1 (not shown) correspont to orbitalas localized at the carborane moieties.

Calculated cartesian coordinates and energies of 3

E(RB3PW91) =	-4376.440898 E_h
Sum of electronic and zero-point Energies=	-4375.565784 E_h
Sum of electronic and thermal Energies=	-4375.516460 E_h
Sum of electronic and thermal Enthalpies=	-4375.515516E_h
Sum of electronic and thermal Free Energies=	-4375.645028 E_h

Ge	0.00006	0.00038	-2.19024
Si	1.46583	-0.00010	-0.41924
Si	-1.46582	0.00002	-0.41933
N	3.00052	1.07868	-0.31232
N	3.00060	-1.07876	-0.31234
N	-3.00041	-1.07883	-0.31202
N	-3.00074	1.07859	-0.31287
C	0.82697	-0.00010	1.41335
C	-0.82706	0.00019	1.41332
C	3.78814	-0.00005	-0.32228
C	5.26867	-0.00000	-0.27468
C	7.43437	0.00055	-1.33987
H	8.03604	0.00076	-2.24399
C	6.04392	0.00023	-1.43606
H	5.56202	0.00019	-2.40842

C	-3.34056	-2.48704	-0.59689
C	3.34085	-2.48722	-0.59570
C	-3.34120	2.48720	-0.59526
C	5.88436	-0.00001	0.98186
H	5.26901	-0.00024	1.87659
C	3.34102	2.48709	-0.59604
C	-5.26867	-0.00024	-0.27459
C	-3.78816	-0.00019	-0.32234
C	-6.04398	0.00063	-1.43595
H	-5.56211	0.00107	-2.40832
C	-5.88427	-0.00100	0.98199
H	-5.26886	-0.00175	1.87667
B	-1.43909	-0.88265	2.74022
H	-2.44978	-1.48889	2.58866
C	8.04900	0.00061	-0.08865
H	9.13270	0.00090	-0.01629
B	1.43892	0.88197	2.74071
H	2.44964	1.48827	2.58957
C	-2.12099	-3.33453	-0.22979
H	-1.22943	-2.97531	-0.75375
H	-2.29818	-4.37428	-0.52065
H	-1.93730	-3.30424	0.84607
B	0.00020	1.41989	1.86501
H	0.00023	2.33289	1.12479
B	1.43864	-0.88304	2.74025
H	2.44918	-1.48955	2.58872
B	-1.43880	0.88233	2.74060
H	-2.44926	1.48906	2.58930
C	2.12107	-3.33432	-0.22842
H	1.93685	-3.30303	0.84732
H	2.29835	-4.37434	-0.51823
H	1.22979	-2.97553	-0.75315
B	-0.00029	-1.42019	1.86447
H	-0.00033	-2.33298	1.12397
C	-4.54108	2.97282	0.22782
H	-5.48247	2.52999	-0.10106
H	-4.62751	4.05872	0.12136
H	-4.39710	2.74289	1.28770
C	-8.04897	0.00022	-0.08842
H	-9.13266	0.00045	-0.01599
C	-4.54075	-2.97408	0.22491
H	-4.39732	-2.74569	1.28520
H	-4.62702	-4.05983	0.11682
H	-5.48203	-2.53089	-0.10376
B	-0.89138	-0.00049	4.18271
H	-1.53761	-0.00050	5.18278
C	7.27228	0.00029	1.07113
H	7.74887	0.00029	2.04695
B	-0.00032	-1.44545	3.62855
H	-0.00046	-2.48399	4.21029
B	0.00014	1.44441	3.62919

H	0.00018	2.48269	4.21138
C	-2.12158	3.33432	-0.22746
H	-1.93755	3.30267	0.84830
H	-2.29893	4.37442	-0.51693
H	-1.23017	2.97583	-0.75215
C	-7.43442	0.00083	-1.33968
H	-8.03614	0.00147	-2.24376
B	0.89116	-0.00073	4.18275
H	1.53735	-0.00098	5.18285
C	-3.61467	-2.62856	-2.10215
H	-4.49984	-2.05704	-2.39440
H	-3.79247	-3.67805	-2.35912
H	-2.75511	-2.26736	-2.67601
C	-7.27219	-0.00074	1.07132
H	-7.74873	-0.00128	2.04716
C	-3.61588	2.63106	-2.10020
H	-2.75643	2.27089	-2.67486
H	-3.79389	3.68094	-2.35541
H	-4.50106	2.05991	-2.39310
C	4.54113	2.97327	0.22638
H	4.39761	2.74373	1.28640
H	4.62733	4.05916	0.11952
H	5.48247	2.53051	-0.10274
C	2.12148	3.33451	-0.22864
H	1.22999	2.97553	-0.75283
H	2.29871	4.37438	-0.51907
H	1.93765	3.30382	0.84719
C	3.61558	-2.63027	-2.10071
H	2.75628	-2.26954	-2.67526
H	3.79333	-3.68004	-2.35655
H	4.50094	-2.05918	-2.39321
C	3.61532	2.62983	-2.10114
H	4.50026	2.05825	-2.39393
H	3.79357	3.67950	-2.35707
H	2.75566	2.26949	-2.67538
C	4.54062	-2.97350	0.22717
H	5.48205	-2.53048	-0.10134
H	4.62704	-4.05933	0.11998
H	4.39655	-2.74429	1.28719

Calculated cartesian coordinates and energies of 4

E(RB3PW91) =	-9952.644008 E_h
Sum of electronic and zero-point Energies=	-9950.900093 E_h
Sum of electronic and thermal Energies=	-9950.794588 E_h
Sum of electronic and thermal Enthalpies=	-9950.793644 E_h
Sum of electronic and thermal Free Energies=	-9951.037059 E_h

Ge	-0.80417	-0.17236	-0.59123
Si	-2.48784	1.53075	-0.48272
Si	-2.10871	-1.61736	0.86983

N	-2.50179	2.76798	-1.87949
N	-2.49206	3.26993	0.21939
N	-1.93785	-3.44997	0.52510
N	-1.46635	-2.48010	2.39175
C	-4.16483	0.89509	-0.24202
C	-3.87997	-1.27669	1.05210
C	-2.63143	3.77555	-1.00915
C	-2.97829	5.17550	-1.33552
C	-1.59059	-0.60210	3.88715
H	-0.90246	-0.06936	3.22160
H	-1.41960	-0.24973	4.90911
H	-2.61978	-0.36160	3.60984
C	-1.33475	-2.11110	3.81376
C	-1.91468	5.38221	1.44653
H	-1.03195	5.51155	0.81966
H	-1.67409	5.73370	2.45426
H	-2.70730	6.02375	1.05974
C	-2.89135	1.17761	-3.64960
H	-1.91809	0.71435	-3.45969
H	-3.13596	1.05301	-4.70949
H	-3.64430	0.64519	-3.06122
B	-4.11649	0.41489	1.33353
H	-3.30220	0.95372	2.03372
C	-1.62018	-4.99397	2.47135
C	-2.87172	2.67279	-3.30304
C	-2.33147	3.91057	1.53878
B	-6.78358	0.45686	0.33678
H	-7.90231	0.89936	0.17707
B	-5.87163	0.58551	1.88583
H	-6.29785	1.14636	2.85363
C	-1.62071	-3.67185	1.80334
C	-4.61034	6.95208	-1.30659
H	-5.60839	7.30901	-1.06938
C	-2.04931	6.03121	-1.92800
H	-1.05322	5.66383	-2.14878
C	-4.26236	5.63506	-1.02123
H	-4.97209	4.95040	-0.56552
C	-2.86087	-5.60279	2.69727
H	-3.76741	-5.08184	2.40278
C	-0.44343	-5.63648	2.85650
H	0.51333	-5.16793	2.66104
C	-1.22108	3.13102	2.25590
H	-1.47799	2.07319	2.35183
H	-1.06604	3.53789	3.25993
H	-0.28009	3.19148	1.70142
C	-3.64198	3.83846	2.33244
H	-4.43027	4.39416	1.81799
H	-3.49182	4.28482	3.32183
H	-3.98319	2.81215	2.46079
C	-2.36235	-2.84197	4.68734
H	-3.36610	-2.72169	4.27003

H	-2.35356	-2.41145	5.69394
H	-2.13871	-3.90736	4.77714
B	-4.33738	-0.80433	-0.45654
H	-3.78227	-1.31282	-1.39846
C	-4.26206	3.26758	-3.56454
H	-4.98984	2.83541	-2.87202
H	-4.57522	3.03342	-4.58745
H	-4.26777	4.35432	-3.45357
C	0.09317	-2.39448	4.29398
H	0.32381	-3.46210	4.24970
H	0.20361	-2.06925	5.33422
H	0.81028	-1.84586	3.67607
B	-6.57092	-0.99656	1.35297
H	-7.54995	-1.50109	1.86223
B	-5.46911	1.58202	0.44463
H	-5.65008	2.76596	0.40319
C	-1.81037	3.36781	-4.16529
H	-1.78727	4.44424	-3.97922
H	-2.03454	3.21581	-5.22652
H	-0.82129	2.95063	-3.95585
C	-3.67885	7.81233	-1.89124
H	-3.95149	8.84181	-2.10609
C	-2.91683	-6.85015	3.31142
H	-3.87994	-7.31809	3.49410
B	-5.03900	-0.96055	2.19192
H	-4.87280	-1.33075	3.31623
C	-1.73945	-7.49675	3.69162
H	-1.78494	-8.47266	4.16679
B	-5.59140	0.50851	-0.95528
H	-5.82733	0.86399	-2.07356
C	-0.50565	-6.88958	3.46238
H	0.41261	-7.39198	3.75277
C	-2.39935	7.35205	-2.19964
H	-1.67122	8.02148	-2.64869
B	-6.16752	-1.08621	-0.40931
H	-6.81517	-1.69688	-1.23244
C	-1.33426	-5.67055	-0.46040
H	-0.28921	-5.48767	-0.20087
H	-1.35686	-6.21053	-1.41239
H	-1.76960	-6.32041	0.30019
K	-9.09412	-1.33537	-0.15574
B	-5.28606	-2.05261	0.82637
H	-5.40553	-3.24112	0.92012
C	-1.52614	-3.60776	-1.83654
H	-2.06830	-2.67735	-2.02467
H	-1.59849	-4.23337	-2.73306
H	-0.47989	-3.33851	-1.66586
C	-3.58383	-4.64525	-0.86499
H	-4.02127	-5.16144	-0.00628
H	-3.69597	-5.28415	-1.74844
H	-4.14060	-3.72030	-1.02609

C	-2.09836	-4.35204	-0.62643
Ge	1.19416	-0.07131	0.95364
Si	2.59683	1.87301	1.00680
Si	2.71501	-1.18866	-0.57337
N	2.41286	2.96629	2.50733
N	2.31166	3.64650	0.46257
C	4.35248	1.54795	0.71672
C	2.37248	4.05554	1.73350
N	2.85418	-3.04031	-0.34015
N	2.21526	-2.05254	-2.14690
C	4.40082	-0.54358	-0.73527
C	2.57000	-3.23517	-1.62976
C	2.78763	2.80322	3.92447
C	2.05919	4.36624	-0.79854
B	5.55386	2.46346	0.14213
B	5.81545	1.28734	1.44018
C	2.49668	5.45817	2.18617
C	3.15406	-3.97404	0.75990
C	2.02662	-1.62250	-3.54391
B	4.37021	1.15977	-0.87289
B	4.79883	-0.14565	0.81814
B	5.51282	0.00445	-1.81642
B	5.90813	-1.12437	-0.51732
C	2.78751	-4.49634	-2.37478
C	3.04590	1.30557	4.13876
C	4.06311	3.58669	4.26257
C	1.62394	3.23894	4.82352
C	1.06787	3.50125	-1.58872
C	3.36755	4.54451	-1.58061
C	1.44124	5.75358	-0.59297
H	5.59072	3.65618	0.27482
B	7.01577	1.51765	0.18541
H	5.96951	1.58771	2.58664
C	1.44492	6.10772	2.83205
C	3.69868	6.13203	1.94097
C	2.60423	-5.38241	0.50521
C	2.47000	-3.40315	2.00576
C	4.66592	-4.04816	0.99905
C	2.02068	-0.09037	-3.51558
C	3.16594	-2.11140	-4.44756
C	0.66893	-2.11113	-4.06116
H	3.50654	1.63264	-1.56353
H	4.29246	-0.79140	1.70063
B	6.08813	1.63225	-1.36597
H	5.42354	-0.28558	-2.97434
B	6.64886	-0.14799	0.79467
H	6.17921	-2.27437	-0.70046
B	7.02872	0.13274	-0.94242
C	4.11306	-4.87943	-2.61318
C	1.73446	-5.29839	-2.81553
H	2.16061	0.71149	3.89096

H	3.30144	1.12756	5.18826
H	3.87923	0.95482	3.52307
H	4.85433	3.34037	3.54880
H	4.40625	3.31271	5.26542
H	3.89396	4.66595	4.25033
H	1.42160	4.30832	4.72569
H	1.86886	3.03827	5.87186
H	0.71889	2.68160	4.56604
H	1.44990	2.48671	-1.73058
H	0.88500	3.94239	-2.57370
H	0.11528	3.42212	-1.05724
H	4.07155	5.15269	-1.00626
H	3.15783	5.05466	-2.52772
H	3.84434	3.58857	-1.79501
H	0.55510	5.71036	0.04058
H	1.14374	6.14390	-1.57108
H	2.14034	6.46727	-0.15517
H	8.05978	2.09261	0.41322
H	0.51511	5.57641	2.99963
C	1.58832	7.43640	3.22558
C	3.84127	7.45498	2.34853
H	4.50777	5.60355	1.44479
H	1.54705	-5.34671	0.23308
H	2.69757	-5.96550	1.42684
H	3.14423	-5.91437	-0.27952
H	2.85936	-2.41312	2.25656
H	2.64075	-4.06257	2.86379
H	1.39421	-3.29177	1.84245
H	5.18219	-4.43525	0.11692
H	4.87153	-4.71764	1.84171
H	5.07562	-3.06260	1.22704
H	1.24315	0.27103	-2.83288
H	1.80518	0.29728	-4.51631
H	2.99010	0.30069	-3.19695
H	4.13332	-1.84793	-4.01064
H	3.08524	-1.62845	-5.42699
H	3.12873	-3.19209	-4.60371
H	0.62269	-3.20295	-4.08941
H	0.50721	-1.74075	-5.07941
H	-0.13077	-1.73231	-3.41791
H	6.39710	2.33536	-2.30484
H	7.40026	-0.69259	1.55000
H	8.07952	-0.19336	-1.45451
H	4.91870	-4.23701	-2.26947
C	4.37815	-6.06255	-3.29601
H	0.71226	-5.00597	-2.60689
C	2.00685	-6.48651	-3.49040
H	0.76388	7.94508	3.71692
H	4.77625	7.97736	2.16717
K	8.92360	2.15491	-1.98302
H	5.40650	-6.35645	-3.48552

H	1.18637	-7.11571	-3.82356
C	2.78573	8.10952	2.98663
H	2.89792	9.14437	3.29773
C	3.32555	-6.86908	-3.73244
H	3.53405	-7.79561	-4.26006

Calculated cartesian coordinates and energies of 5 ($[B\{C_6H_3(CF_3)_2\}_4]^-$ counter ions are not included)

E(RB3PW91) =	-8752.561344 E_h
Sum of electronic and zero-point Energies=	-8750.806418 E_h
Sum of electronic and thermal Energies=	-8750.707394 E_h
Sum of electronic and thermal Enthalpies=	-8750.706450 E_h
Sum of electronic and thermal Free Energies=	-8750.932916 E_h

Ge	-0.11017	1.15677	-0.68938
Si	2.10569	2.02697	-0.40091
N	3.59862	1.48172	0.49177
C	2.06645	3.94627	-0.27448
B	1.85425	4.57800	1.29608
H	1.80931	3.81912	2.19297
Ge	-0.80168	-0.43727	1.18403
Si	-0.76442	3.20966	0.38174
N	3.42233	1.71861	-1.64694
C	0.58469	4.55465	0.15519
B	3.32446	4.90477	0.37092
H	4.32077	4.35007	0.69162
Si	0.23689	-2.59676	1.02045
N	-1.67230	3.63770	1.91629
C	-1.10579	-3.96740	0.84369
B	2.87708	5.02326	-1.32909
H	3.58327	4.55886	-2.15463
Si	-2.59621	-1.50401	-0.01071
N	-2.30282	4.07381	-0.10406
C	-2.55394	-3.40109	0.26586
B	1.12692	4.75301	-1.45392
H	0.61875	4.09944	-2.29789
N	1.31934	-3.24309	2.35652
C	4.28921	1.43607	-0.66623
B	0.31047	6.07126	-0.58520
H	-0.80117	6.34139	-0.88909
N	1.75616	-3.24384	0.24217
C	-2.62444	4.24565	1.18587
B	0.75443	5.95254	1.11952
H	-0.05426	6.13035	1.96858
N	-4.34518	-1.07635	0.31809
C	2.20777	-3.65756	1.44387
B	2.50981	6.21244	1.26253
H	2.98544	6.67540	2.24699
N	-3.41822	-1.23210	-1.62749
C	-4.58707	-1.01409	-0.99913

B	3.14308	6.49923	-0.38662
H	4.09302	7.18173	-0.59068
C	5.75154	1.26346	-0.81260
B	1.77376	6.39988	-1.53623
H	1.72196	6.99986	-2.55943
C	6.34388	0.03506	-1.11533
H	5.73562	-0.85610	-1.21410
B	1.55289	7.14922	0.07555
H	1.33701	8.30894	0.20994
C	7.72430	-0.04011	-1.28877
H	8.18465	-0.99455	-1.52461
B	-1.55771	-4.33427	-0.75941
H	-0.98448	-3.79436	-1.63322
C	8.51020	1.10477	-1.16464
H	9.58486	1.04289	-1.30514
B	-2.21986	-5.96637	-0.72420
H	-2.11113	-6.66414	-1.67878
C	7.91666	2.33059	-0.86136
H	8.52543	3.22430	-0.76684
B	-2.10796	-6.55275	0.96256
H	-1.91450	-7.69441	1.22487
C	6.53907	2.41498	-0.68472
H	6.06871	3.36781	-0.46290
B	-1.36060	-5.26805	1.92950
H	-0.61040	-5.39092	2.83457
C	3.59620	1.65135	-3.12469
B	-0.80968	-5.55250	0.28091
H	0.31681	-5.85029	0.06858
C	3.61295	0.17464	-3.53379
H	4.45035	-0.35359	-3.07248
H	3.71965	0.09028	-4.61921
H	2.68084	-0.31100	-3.23927
B	-3.65794	-5.93896	0.33921
H	-4.60171	-6.63312	0.14627
C	4.87989	2.34477	-3.59636
H	4.96603	3.35132	-3.18190
H	4.84867	2.42903	-4.68620
H	5.77602	1.78122	-3.33327
B	-3.12191	-5.49382	1.98960
H	-3.66233	-5.85441	2.98339
C	2.38301	2.32957	-3.76617
H	1.44243	1.91641	-3.38461
H	2.40739	2.15752	-4.84539
H	2.39150	3.40722	-3.60206
B	-2.44104	-3.86139	1.90960
H	-2.42626	-3.01018	2.73030
C	4.01058	1.09663	1.86224
B	-3.31272	-4.56053	-0.72859
H	-3.94072	-4.15853	-1.65080
C	5.10451	2.01996	2.40717
H	6.02796	1.93864	1.83171

H	5.32766	1.74492	3.44220
H	4.77341	3.06074	2.39580
B	-3.86217	-4.27984	0.92672
H	-4.87226	-3.69455	1.12146
C	4.47753	-0.36245	1.86258
H	3.66910	-1.01496	1.51730
H	4.74456	-0.65363	2.88243
H	5.35385	-0.51192	1.22904
C	2.76775	1.20390	2.74351
H	2.42068	2.23368	2.81046
H	3.00660	0.87071	3.75614
H	1.95108	0.58380	2.35670
C	-3.72946	5.08168	1.69854
C	-3.45567	6.44392	1.87664
H	-2.46524	6.82885	1.65332
C	-4.45768	7.28983	2.33956
H	-4.24770	8.34560	2.47926
C	-5.72737	6.78297	2.62211
H	-6.50716	7.44637	2.98307
C	-5.99623	5.42712	2.44086
H	-6.98401	5.03326	2.65926
C	-4.99819	4.57140	1.97778
H	-5.20689	3.51819	1.83190
C	-1.64654	3.37022	3.37914
C	-0.27650	2.78272	3.71001
H	-0.04056	1.93289	3.06037
H	-0.27487	2.42182	4.74172
H	0.50409	3.53916	3.61884
C	-2.72834	2.33324	3.70294
H	-3.72516	2.71556	3.47275
H	-2.70239	2.08840	4.76893
H	-2.55571	1.41342	3.13571
C	-1.85472	4.64663	4.20196
H	-1.16532	5.43273	3.88350
H	-1.65395	4.42373	5.25385
H	-2.87527	5.02468	4.13237
C	-3.12623	4.29631	-1.31897
C	-2.21505	4.12055	-2.53486
H	-1.48639	4.92973	-2.60023
H	-2.81909	4.13819	-3.44570
H	-1.68182	3.16465	-2.50472
C	-3.75337	5.69454	-1.36178
H	-4.53831	5.82390	-0.61594
H	-4.20159	5.84516	-2.34810
H	-2.99787	6.46931	-1.21229
C	-4.22051	3.22136	-1.33705
H	-3.77745	2.22259	-1.27059
H	-4.79128	3.28958	-2.26835
H	-4.91282	3.35617	-0.50222
C	3.37785	-4.52774	1.69569
C	3.17420	-5.91031	1.59899

H	2.19585	-6.29438	1.32749
C	4.22363	-6.78209	1.87340
H	4.06328	-7.85334	1.80312
C	5.47230	-6.28130	2.24296
H	6.28782	-6.96431	2.45949
C	5.67284	-4.90470	2.33655
H	6.64365	-4.51326	2.62437
C	4.62763	-4.02434	2.06560
H	4.78648	-2.95505	2.13711
C	1.39104	-3.27136	3.84370
C	2.41579	-2.22445	4.29366
H	3.41670	-2.47403	3.93479
H	2.45138	-2.18518	5.38628
H	2.14053	-1.23585	3.92033
C	1.77719	-4.65357	4.38394
H	1.14790	-5.43860	3.95946
H	1.63563	-4.65576	5.46834
H	2.82161	-4.89851	4.18613
C	0.01055	-2.88007	4.37653
H	-0.32560	-1.92405	3.95973
H	0.06451	-2.76917	5.46258
H	-0.73505	-3.64461	4.15693
C	2.40008	-3.30844	-1.09202
C	2.50938	-4.75040	-1.59607
H	3.15089	-5.35390	-0.95152
H	2.94425	-4.75210	-2.59988
H	1.52443	-5.21896	-1.65012
C	3.78063	-2.64683	-1.03180
H	3.68383	-1.61225	-0.68917
H	4.21833	-2.64032	-2.03421
H	4.46557	-3.17966	-0.37013
C	1.52144	-2.49875	-2.04262
H	0.53318	-2.94442	-2.13882
H	1.97359	-2.48405	-3.03688
H	1.40673	-1.46524	-1.69803
C	-5.91726	-0.89156	-1.63049
C	-6.63318	-2.08151	-1.81598
H	-6.19593	-3.02664	-1.50858
C	-7.89824	-2.03787	-2.39170
H	-8.45437	-2.95851	-2.53794
C	-8.45057	-0.81497	-2.77693
H	-9.43904	-0.78454	-3.22469
C	-7.73632	0.36697	-2.58686
H	-8.16677	1.31792	-2.88499
C	-6.46666	0.33306	-2.01285
H	-5.91201	1.25131	-1.86052
C	-5.19473	-0.63076	1.45084
C	-4.51839	-1.08397	2.74595
H	-4.55026	-2.16989	2.84680
H	-5.04660	-0.65177	3.59971
H	-3.47536	-0.75530	2.79660

C	-6.60987	-1.21853	1.39749
H	-7.20487	-0.80716	0.58146
H	-7.12104	-0.98246	2.33529
H	-6.58004	-2.30561	1.29425
C	-5.25324	0.90143	1.40478
H	-4.24335	1.32352	1.39038
H	-5.77939	1.27988	2.28660
H	-5.78936	1.24208	0.51525
C	-3.09623	-1.11393	-3.07447
C	-1.66212	-1.60579	-3.25465
H	-0.98492	-1.11180	-2.54878
H	-1.31783	-1.37186	-4.26530
H	-1.60253	-2.68699	-3.12250
C	-4.03095	-1.96003	-3.94600
H	-4.05881	-2.99588	-3.59848
H	-3.65248	-1.95661	-4.97232
H	-5.04859	-1.56882	-3.96626
C	-3.17227	0.36394	-3.47516
H	-4.18181	0.75904	-3.34543
H	-2.90273	0.47546	-4.52965
H	-2.47327	0.95784	-2.87873

Calculated cartesian coordinates and energies of 7 singlet

E(RB3PW91) =	-8752.877100 E_h
Sum of electronic and zero-point Energies=	-8751.131148 E_h
Sum of electronic and thermal Energies=	-8751.029868 E_h
Sum of electronic and thermal Enthalpies=	-8751.028923 E_h
Sum of electronic and thermal Free Energies=	-8751.259163 E_h

Ge	0.54475	0.30557	1.10140
Ge	-1.32857	0.71188	-0.59488
Si	1.62205	2.37937	0.74585
Si	-2.69753	2.37525	0.48517
Si	-2.38564	-1.37350	-0.22014
Si	1.92762	-1.34474	0.03015
N	3.62152	-1.28492	-0.72302
N	-4.38833	2.31164	1.24415
N	3.35194	2.68376	1.31076
N	3.15197	-2.16435	1.18624
N	-4.10415	-1.69634	-0.80657
N	-3.92481	3.20769	-0.65867
N	2.58270	3.23735	-0.62319
N	-3.35793	-2.22306	1.14486
C	-1.33316	-2.71191	-0.94714
C	0.58041	3.70844	1.49812
C	1.07620	-2.72489	-0.85425
C	-1.83434	3.74393	1.37214
C	-5.60948	-3.20255	0.53131
C	4.84495	4.20437	-0.02740
C	3.64008	3.35944	0.18871

C	4.12717	-2.00659	0.28271
C	-4.40432	-2.35803	0.32077
C	-4.89889	3.03571	0.24261
C	-4.99940	1.77702	2.47011
C	5.46827	-2.63918	0.28432
B	-0.70649	3.32832	2.58973
H	-0.75135	2.22599	3.04732
B	-0.75399	4.77946	3.74474
H	-0.82012	4.69760	4.93279
B	-0.07124	-2.33289	-2.06481
H	-0.04355	-1.23578	-2.53570
B	-0.15390	-3.54926	-0.01840
H	-0.18053	-3.45142	1.16797
C	4.23090	-0.76768	-1.95734
C	-6.24801	3.65066	0.23951
B	1.42835	-3.41990	-2.29001
H	2.41326	-3.07530	-2.85694
B	-0.57868	4.56097	0.56664
H	-0.52857	4.47035	-0.61791
C	-5.79162	2.82545	3.26101
H	-5.19881	3.73019	3.40903
H	-6.04118	2.41313	4.24374
H	-6.72555	3.09411	2.76315
C	6.62251	-1.95961	0.68043
H	6.55075	-0.94924	1.06828
C	-4.94931	-1.31692	-1.95197
C	-5.54624	-4.53325	0.09875
H	-4.63424	-4.89984	-0.36161
C	6.01634	3.72203	-0.61723
H	6.06509	2.69962	-0.96952
C	4.19913	2.31709	2.45774
C	1.10847	3.07911	-2.52329
H	1.29843	2.01404	-2.66974
H	0.82375	3.51348	-3.48600
H	0.25738	3.17532	-1.84803
C	-6.78732	-2.71564	1.10388
H	-6.83883	-1.69146	1.45104
B	-0.02635	-3.79714	-3.20263
H	0.02120	-3.72748	-4.39224
B	-2.20087	4.43148	2.80739
H	-3.19935	4.09550	3.35587
C	-1.89304	-2.05760	3.04891
H	-1.04303	-2.15810	2.37273
H	-1.60875	-2.48870	4.01316
H	-2.08092	-0.99184	3.19086
C	-7.38637	2.96505	-0.18946
H	-7.29519	1.96282	-0.59426
C	-2.85450	-4.29510	2.44778
H	-3.70670	-4.83101	2.02263
H	-2.70009	-4.66254	3.46773
H	-1.96825	-4.53082	1.85928

C	-4.04586	-0.60608	-2.96517
H	-3.56352	0.26914	-2.51940
H	-4.64547	-0.27543	-3.81859
H	-3.26002	-1.26832	-3.33398
C	-3.12080	-2.78502	2.49463
C	5.15179	0.39812	-1.58393
H	5.55924	0.86320	-2.48784
H	5.98879	0.06019	-0.96771
H	4.58185	1.14543	-1.02698
C	3.31173	1.54163	3.43668
H	3.90557	1.22891	4.30082
H	2.89501	0.64894	2.96105
H	2.48002	2.15279	3.79423
C	-4.31668	-2.51396	3.41777
H	-4.59180	-1.45891	3.39858
H	-4.03953	-2.77473	4.44380
H	-5.18842	-3.11222	3.14678
C	5.01423	-1.82968	-2.73875
H	4.41604	-2.73279	-2.87411
H	5.94800	-2.09784	-2.24019
H	5.26329	-1.43056	-3.72701
C	4.78665	5.53314	0.41176
H	3.88011	5.89719	0.88452
C	3.52822	3.53703	-2.90124
H	4.40148	4.13521	-2.63531
H	3.24365	3.80049	-3.92456
H	3.80428	2.48183	-2.88716
C	-6.35996	4.95015	0.74918
H	-5.46642	5.46627	1.08836
C	2.07556	5.31501	-1.90979
H	1.20204	5.54948	-1.30208
H	1.90260	5.68915	-2.92432
H	2.93746	5.84584	-1.49788
C	3.07068	-0.23445	-2.80237
H	2.40650	-1.03677	-3.12676
H	3.45382	0.27406	-3.69143
H	2.48614	0.48370	-2.21847
C	5.88950	6.36796	0.25400
H	5.83392	7.39661	0.59738
B	-1.56187	6.08568	2.80871
H	-2.19627	6.96696	3.30425
C	5.31753	1.38583	1.97002
H	5.99964	1.90162	1.28968
H	4.87841	0.53136	1.44622
H	5.89897	1.01594	2.82095
C	-6.65195	-5.36598	0.24597
H	-6.59356	-6.39622	-0.09210
B	0.77991	4.37760	2.97445
H	1.69790	4.01485	3.63915
C	5.55587	-3.95056	-0.19833
H	4.65240	-4.45984	-0.52116

C	-3.83912	1.24364	3.31409
H	-3.24734	0.53642	2.72432
H	-4.22160	0.72293	4.19630
H	-3.18242	2.04781	3.64900
B	0.88103	5.30528	1.45773
H	1.87868	5.66504	0.91721
B	-1.61740	-4.31265	-0.88267
H	-2.60522	-4.67577	-0.32751
C	2.33810	3.80458	-1.96919
B	0.81047	-5.08309	-2.26494
H	1.44810	-5.96226	-2.75993
C	-4.28335	5.33524	-1.92164
H	-5.26468	5.53847	-1.48855
H	-4.27121	5.75596	-2.93232
H	-3.52364	5.84477	-1.32617
C	-5.91304	0.61082	2.08021
H	-6.74440	0.95120	1.45772
H	-6.32869	0.13939	2.97705
H	-5.33637	-0.13199	1.52407
B	1.37959	-4.32365	-0.76551
H	2.32652	-4.68238	-0.14033
C	3.21611	-2.77857	2.53309
C	-6.01889	-0.32790	-1.46730
H	-6.70671	-0.80066	-0.76136
H	-6.60196	0.04494	-2.31576
H	-5.53827	0.52184	-0.97343
B	-1.55086	-3.40468	-2.41100
H	-2.48730	-3.06634	-3.06161
B	0.18986	6.05646	2.90927
H	0.79307	6.91972	3.47122
C	-5.59348	-2.54025	-2.61564
H	-4.83083	-3.28800	-2.84908
H	-6.07614	-2.23463	-3.54920
H	-6.35310	-2.99854	-1.97946
C	-5.07380	3.13446	-2.84145
H	-4.89552	2.05838	-2.88353
H	-5.05174	3.52670	-3.86303
H	-6.07392	3.31087	-2.43915
C	7.11710	4.56211	-0.77084
H	8.02209	4.17930	-1.23361
B	-2.11541	5.34793	1.29098
H	-3.04691	5.72336	0.65150
C	7.86128	-2.59166	0.59423
H	8.75699	-2.06289	0.90674
C	3.33092	-4.30620	2.45485
H	2.46927	-4.73562	1.94087
H	3.37139	-4.71682	3.46906
H	4.23917	-4.61461	1.93270
B	-0.94196	-5.07449	-2.33765
H	-1.54618	-5.94922	-2.88029
C	-7.89110	-3.55380	1.24646

H	-8.80183	-3.16813	1.69539
C	-7.82625	-4.87891	0.81915
H	-8.68815	-5.53008	0.93237
C	-8.63513	3.57867	-0.10776
H	-9.51905	3.04518	-0.44481
C	-3.99431	3.83060	-2.00117
C	4.77694	3.55399	3.15597
H	5.51756	4.06288	2.53582
H	5.26720	3.25197	4.08681
H	3.97713	4.25861	3.39847
B	-0.12851	-5.25105	-0.74373
H	-0.16176	-6.25934	-0.10632
C	-7.61019	5.55580	0.82932
H	-7.69406	6.56201	1.22876
C	-8.74873	4.87087	0.40296
H	-9.72376	5.34497	0.46802
C	1.91733	-2.42065	3.25788
H	1.79661	-1.34073	3.37567
H	1.91701	-2.88227	4.24993
H	1.05300	-2.80126	2.70913
C	-2.63061	3.62781	-2.66364
H	-1.84734	4.12372	-2.08516
H	-2.64301	4.06906	-3.66470
H	-2.37461	2.56811	-2.75333
C	7.95057	-3.89656	0.11074
H	8.91830	-4.38484	0.04180
C	7.05656	5.88531	-0.33712
H	7.91597	6.53814	-0.45923
C	6.79704	-4.57477	-0.28349
H	6.86140	-5.58994	-0.66341
C	4.40324	-2.20507	3.32013
H	5.36009	-2.54165	2.91639
H	4.34469	-2.54129	4.35992
H	4.38180	-1.11297	3.31199
B	-0.59732	6.25786	1.30470
H	-0.54343	7.27173	0.67749

Calculated cartesian coordinates and energies of 7 triplet

E(RB3PW91) =	-8752.902687 E_h
Sum of electronic and zero-point Energies=	-8751.155693 E_h
Sum of electronic and thermal Energies=	-8751.054735E_h
Sum of electronic and thermal Enthalpies=	-8751.053791 E_h
Sum of electronic and thermal Free Energies=	-8751.284342 E_h

Ge	0.53945	0.30313	1.10897
Ge	-1.32301	0.71514	-0.60499
Si	1.60875	2.38325	0.73994
Si	-2.69226	2.37860	0.47240
Si	-2.37480	-1.37492	-0.21835
Si	1.92365	-1.34907	0.04180
N	3.61054	-1.29302	-0.72510

N	-4.37617	2.32161	1.24489
N	3.33099	2.70187	1.31639
N	3.16080	-2.15416	1.19710
N	-4.08877	-1.70800	-0.80944
N	-3.93067	3.19876	-0.67091
N	2.57803	3.23121	-0.63156
N	-3.35208	-2.21478	1.15153
C	-1.31906	-2.72266	-0.93754
C	0.56297	3.72575	1.48062
C	1.07174	-2.73802	-0.83584
C	-1.82887	3.75803	1.35286
C	-5.59968	-3.20166	0.53438
C	4.83418	4.20673	-0.02610
C	3.62681	3.36508	0.18901
C	4.12611	-2.00405	0.28294
C	-4.39273	-2.35983	0.32240
C	-4.89563	3.03502	0.24047
C	-4.98203	1.78257	2.47168
C	5.47052	-2.62962	0.27940
B	-0.70792	3.35296	2.58894
H	-0.75053	2.25324	3.05326
B	-0.75878	4.80663	3.72402
H	-0.82594	4.73335	4.91274
B	-0.06660	-2.35120	-2.06414
H	-0.03738	-1.25540	-2.53882
B	-0.15100	-3.57136	-0.00037
H	-0.18285	-3.47298	1.18581
C	4.21474	-0.76990	-1.95961
C	-6.24724	3.64444	0.24132
B	1.43583	-3.43946	-2.26767
H	2.42703	-3.10020	-2.82669
B	-0.58619	4.58789	0.54089
H	-0.53460	4.49546	-0.64355
C	-5.78090	2.82539	3.26332
H	-5.19464	3.73453	3.41025
H	-6.02574	2.41125	4.24647
H	-6.71770	3.08688	2.76702
C	6.62080	-1.94549	0.67861
H	6.54340	-0.93691	1.07022
C	-4.93454	-1.32642	-1.95417
C	-5.54057	-4.53220	0.10114
H	-4.63025	-4.90108	-0.36062
C	6.00416	3.72054	-0.61533
H	6.04957	2.69811	-0.96814
C	4.17810	2.32863	2.46209
C	1.10238	3.07993	-2.53086
H	1.28383	2.01254	-2.67129
H	0.82245	3.51138	-3.49626
H	0.25147	3.18745	-1.85712
C	-6.77507	-2.71136	1.10895
H	-6.82305	-1.68702	1.45622

B	-0.01766	-3.81426	-3.18730
H	0.03433	-3.75044	-4.37732
B	-2.20501	4.45366	2.78373
H	-3.20649	4.12029	3.32799
C	-1.88314	-2.05614	3.05340
H	-1.03318	-2.16652	2.37878
H	-1.60292	-2.48448	4.02006
H	-2.06387	-0.98839	3.19003
C	-7.38311	2.95469	-0.18732
H	-7.28853	1.95284	-0.59243
C	-2.85919	-4.28902	2.45671
H	-3.71645	-4.82065	2.03646
H	-2.70286	-4.65517	3.47681
H	-1.97705	-4.53158	1.86507
C	-4.02959	-0.62278	-2.97102
H	-3.54339	0.25252	-2.52942
H	-4.62897	-0.29303	-3.82494
H	-3.24679	-1.28960	-3.33809
C	-3.11578	-2.77726	2.50183
C	5.12733	0.40208	-1.58528
H	5.53421	0.86867	-2.48860
H	5.96494	0.06946	-0.96693
H	4.55193	1.14685	-1.03042
C	3.28508	1.57061	3.44965
H	3.87953	1.25392	4.31192
H	2.85198	0.68139	2.98188
H	2.46460	2.19566	3.80910
C	-4.30809	-2.49730	3.42681
H	-4.57034	-1.43904	3.41417
H	-4.03353	-2.76726	4.45115
H	-5.18695	-3.08364	3.15295
C	5.00639	-1.82460	-2.74262
H	4.41631	-2.73316	-2.87683
H	5.94393	-2.08397	-2.24649
H	5.24929	-1.42277	-3.73133
C	4.77963	5.53518	0.41414
H	3.87391	5.90186	0.88637
C	3.52477	3.51924	-2.91077
H	4.40589	4.10362	-2.64025
H	3.24524	3.79257	-3.93288
H	3.78560	2.46036	-2.90279
C	-6.36297	4.94308	0.75204
H	-5.47088	5.46224	1.09036
C	2.08586	5.31050	-1.92411
H	1.21429	5.55350	-1.31720
H	1.91530	5.68229	-2.93988
H	2.95167	5.83672	-1.51453
C	3.04925	-0.24658	-2.80328
H	2.39158	-1.05487	-3.12613
H	3.42681	0.26456	-3.69319
H	2.45945	0.46694	-2.21877

C	5.88520	6.36649	0.25731
H	5.83272	7.39520	0.60097
B	-1.56933	6.10764	2.78232
H	-2.20269	6.99056	3.27608
C	5.28247	1.38118	1.97333
H	5.96654	1.88587	1.28655
H	4.83178	0.52820	1.45685
H	5.86452	1.00927	2.82288
C	-6.64820	-5.36196	0.25044
H	-6.59309	-6.39237	-0.08765
B	0.77484	4.40983	2.95471
H	1.69963	4.05140	3.61187
C	5.56437	-3.93815	-0.20955
H	4.66317	-4.45092	-0.53313
C	-3.81676	1.25826	3.31431
H	-3.22051	0.55473	2.72438
H	-4.19406	0.73572	4.19763
H	-3.16565	2.06769	3.64742
B	0.87373	5.32283	1.43562
H	1.87298	5.67539	0.89397
B	-1.61538	-4.32326	-0.87303
H	-2.60703	-4.67974	-0.32056
C	2.33717	3.79808	-1.97907
B	0.81616	-5.09981	-2.24518
H	1.45121	-5.98144	-2.73908
C	-4.30642	5.32259	-1.93634
H	-5.28890	5.51990	-1.50309
H	-4.29771	5.74153	-2.94778
H	-3.54986	5.83841	-1.34226
C	-5.88857	0.61066	2.08244
H	-6.72134	0.94593	1.45903
H	-6.30244	0.13811	2.97945
H	-5.30760	-0.12999	1.52778
B	1.37970	-4.34117	-0.74599
H	2.32934	-4.69214	-0.12034
C	3.23301	-2.76856	2.54339
C	-5.99836	-0.33144	-1.46903
H	-6.68646	-0.79985	-0.76042
H	-6.58198	0.04220	-2.31672
H	-5.51297	0.51766	-0.97851
B	-1.54419	-3.42621	-2.40156
H	-2.48389	-3.08881	-3.04795
B	0.18389	6.08388	2.87963
H	0.78139	6.95398	3.43690
C	-5.58606	-2.54816	-2.61346
H	-4.82758	-3.30062	-2.84528
H	-6.06783	-2.24249	-3.54746
H	-6.34774	-3.00033	-1.97539
C	-5.08315	3.11472	-2.85072
H	-4.89609	2.04017	-2.89307
H	-5.06731	3.50696	-3.87241

H	-6.08345	3.28308	-2.44531
C	7.10808	4.55689	-0.76686
H	8.01239	4.17128	-1.22862
B	-2.11913	5.36512	1.26800
H	-3.05576	5.72812	0.62889
C	7.86301	-2.57043	0.58873
H	8.75611	-2.03830	0.90297
C	3.38376	-4.29306	2.46629
H	2.53810	-4.74166	1.94226
H	3.42130	-4.70269	3.48098
H	4.30491	-4.58134	1.95544
B	-0.93766	-5.09236	-2.31951
H	-1.53556	-5.97180	-2.86149
C	-7.88113	-3.54634	1.25259
H	-8.79025	-3.15818	1.70262
C	-7.82039	-4.87161	0.82516
H	-8.68395	-5.52038	0.93944
C	-8.63404	3.56370	-0.10392
H	-9.51640	3.02718	-0.44021
C	-4.00708	3.81996	-2.01376
C	4.77555	3.56087	3.15200
H	5.52228	4.05572	2.52790
H	5.26341	3.25627	4.08323
H	3.98647	4.27809	3.39271
B	-0.12653	-5.26514	-0.72352
H	-0.16466	-6.27333	-0.08586
C	-7.61524	5.54427	0.83302
H	-7.70247	6.55006	1.23276
C	-8.75167	4.85532	0.40735
H	-9.72832	5.32592	0.47344
C	1.92263	-2.43980	3.26103
H	1.77543	-1.36232	3.37242
H	1.92943	-2.89575	4.25562
H	1.07058	-2.84505	2.71046
C	-2.64296	3.62455	-2.67767
H	-1.86209	4.12696	-2.10152
H	-2.65957	4.06296	-3.67990
H	-2.38039	2.56619	-2.76496
C	7.95880	-3.87266	0.09946
H	8.92907	-4.35552	0.02795
C	7.05134	5.88007	-0.33258
H	7.91311	6.53005	-0.45328
C	6.80873	-4.55540	-0.29735
H	6.87843	-5.56866	-0.68141
C	4.40269	-2.16657	3.33494
H	5.36865	-2.46666	2.92397
H	4.35853	-2.51770	4.37054
H	4.34538	-1.07594	3.34057
B	-0.60588	6.27698	1.27522
H	-0.55163	7.28950	0.64568

C. References

1. Y.-P. Zhou, S. Raoufmoghaddam, T. Szilvási, M. Driess, *Angew. Chem. Int. Ed.* **2016**, *55*, 12868–12872.
2. (a) G. M. Sheldrick, *SHELX-97 Program for Crystal Structure Determination*, Universität Göttingen (Germany) **1997**; (b) S. Stoll, A. Schweiger, *J. Magn. Reson.* 2006, 178, 42-55.
3. Gaussian 09, Revision D.01, M. J. Frisch, G. W. Trucks, H. B. Schlegel, G. E. Scuseria, M. A. Robb, J. R. Cheeseman, G. Scalmani, V. Barone, G. A. Petersson, H. Nakatsuji, X. Li, M. Caricato, A. Marenich, J. Bloino, B. G. Janesko, R. Gomperts, B. Mennucci, H. P. Hratchian, J. V. Ortiz, A. F. Izmaylov, J. L. Sonnenberg, D. Williams-Young, F. Ding, F. Lipparini, F. Egidi, J. Goings, B. Peng, A. Petrone, T. Henderson, D. Ranasinghe, V. G. Zakrzewski, J. Gao, N. Rega, G. Zheng, W. Liang, M. Hada, M. Ehara, K. Toyota, R. Fukuda, J. Hasegawa, M. Ishida, T. Nakajima, Y. Honda, O. Kitao, H. Nakai, T. Vreven, K. Throssell, J. A. Montgomery, Jr., J. E. Peralta, F. Ogliaro, M. Bearpark, J. J. Heyd, E. Brothers, K. N. Kudin, V. N. Staroverov, T. Keith, R. Kobayashi, J. Normand, K. Raghavachari, A. Rendell, J. C. Burant, S. S. Iyengar, J. Tomasi, M. Cossi, J. M. Millam, M. Klene, C. Adamo, R. Cammi, J. W. Ochterski, R. L. Martin, K. Morokuma, O. Farkas, J. B. Foresman, and D. J. Fox, Gaussian, Inc., Wallingford CT, **2016**.
4. A.D. Becke, J. Chem. Phys. 98, 5648 (1993).
5. (a) S.Grimme, S.Ehrlich, L.Goerigk, *J Comput Chem*, (**2011**), 32, 1456–1465; (b) S.Grimme, J.Antony, S.Ehrlich and H.Krieg, *J.Chem.Phys*., 132, (**2010**), 154104.
6. (a) R. Ditchfield, W. J. Hehre, and J. A. Pople, *J. Chem. Phys.*, **54** (1971) 724; (b) W. J. Hehre, R. Ditchfield, and J. A. Pople, *J. Chem. Phys.*, **56** (1972) 2257; (c) P. C. Hariharan and J. A. Pople, *Theor. Chem. Acc.*, **28** (1973) 213-22; (d) P. C. Hariharan and J. A. Pople, *Mol. Phys.*, **27** (1974) 209-14; (e) M. M. Francl, W. J. Pietro, W. J. Hehre, J. S. Binkley, D. J. DeFrees, J. A. Pople, and M. S. Gordon, *J. Chem. Phys.*, **77** (1982) 3654-65; (f) J.-P. Blaudeau, M. P. McGrath, L. A. Curtiss, and L. Radom, *J. Chem. Phys.*, **107** (1997) 5016-21; (g) A. J. H. Wachters, *J. Chem. Phys.*, **52** (1970) 1033; (h) L. A. Curtiss, M. P. McGrath, J.-P. Blaudeau, N. E. Davis, R. C. Binning Jr., and L. Radom, *J. Chem. Phys.*, **103** (1995) 6104-13.
7. NBO 6.0. E. D. Glendening, J. K. Badenhoop, A. E. Reed, J. E. Carpenter, J. A. Bohmann, C. M. Morales, C. R. Landis, and F. Weinhold (Theoretical Chemistry Institute, University of Wisconsin, Madison, WI, **2013**); http://nbo6.chem.wisc.edu/
